# Inhibition of EZH2 alleviates SAHA-induced senescence-associated secretion phenotype in small cell lung cancer cells

**DOI:** 10.1038/s41420-023-01591-y

**Published:** 2023-08-05

**Authors:** Sun-Hyok Kong, Lie Ma, Qingxia Yuan, Xiangxiang Liu, Yu Han, Weifang Xiang, Dong-Xu Liu, Yu Zhang, Jun Lu

**Affiliations:** 1 The Key Laboratory of Molecular Epigenetics of Ministry of Education (MOE), Northeast Normal University, Changchun, 130024 China; 2 School of Life Science, University of Science, Pyongyang, 999091 Democratic People’s Republic of Korea; 3 The Institute of Genetics and Cytology, Northeast Normal University, Changchun, 130024 China; 4 The Centre for Biomedical and Chemical Sciences, School of Science, Faculty of Health and Environmental Sciences, Auckland University of Technology, Auckland, 1010 New Zealand

**Keywords:** Small-cell lung cancer, Senescence

## Abstract

Chemotherapy has been widely used in small cell lung cancer (SCLC) treatment in the past decades. However, SCLC is easy to recur after chemotherapy. The senescence of cancer cells during chemotherapy is one of the effective therapeutic strategies to inhibit the progression of cancer. Nevertheless, the senescence-associated secretion phenotype (SASP) promotes chronic inflammation of the cancer microenvironment and further accelerates the progression of tumors. Therefore, inducing the senescence of cancer cells and inhibiting the production of SASP factors during anticancer treatment have become effective therapeutic strategies to improve the anticancer effect of drugs. Here we reported that SCLC cells treated with an FDA-approved HDAC inhibitor SAHA underwent senescence and displayed remarkable SASP. In particular, SAHA promoted the formation of cytoplasmic chromatin fragments (CCFs) in SCLC cells. The increased CCFs in SAHA-treated SCLC cells were related to nuclear porin Tpr, which activated the cGAS-STING pathway, and promoted the secretion of SASP in cancer cells. Inhibition of EZH2 suppressed the increase of CCFs in SAHA-treated SCLC cells, weakened the production of SASP, and increased the antiproliferative effect of SAHA. Overall, our work affords new insight into the secretion of SASP in SCLC and establishes a foundation for constructing a new therapeutic strategy for SCLC patients.

## Introduction

Lung cancer has the highest incidence and mortality among cancers. According to its characteristics in growth and spread, it is divided into two main classes: non-small cell lung cancer (NSCLC) and small cell lung cancer (SCLC) [1]. A majority of lung cancers are NSCLC. SCLC only accounts for about 10 to 15% of all lung cancers. However, SCLC is characterized by high malignancy and invasiveness, rapid progression of tumors, and early development of widespread metastases [2]. Treatment methods for NSCLC at different stages have been established and are constantly being updated, but there has been no clear progress in the treatment method for SCLC in the last over 30 years [1–3]. In particular, SCLC patients respond sensitively to chemotherapy in the early stages, but in the vast majority of patients, this effect is temporary, and either acquires drug resistance within 2 years or it recurs because of internal factors, resulting in death [4, 5].

Many cancer treatment drugs can cause senescence in different cancer cells during the treatment process [6], and cells display senescence-associated secretory phenotype (SASP) [7, 8]. Cellular senescence can be an effective cancer treatment to suppress the risk of malignant metastasis, but it may also impair the suppression of tumors because senescent cells continuously secrete SASP factors [9–11]. SASP factors promote not only the proliferation and metastasis of cancer cells but also angiogenesis in the tumor microenvironment, and play a critical role in providing a favorable environment for the proliferation of metastasized cancer cells, thus contributing to tolerance for chemotherapy. Therefore, therapies that target SASP may be more beneficial to overcome chemotherapy resistance induced by SASP, and improve the effectiveness of chemotherapy [12]. However, the mechanism by which anticancer drugs promote SASP secretion in cancer cells remains unclear.

Cytoplasmic chromatin fragments (CCFs) formed in the cytoplasm during multiple models of senescence [13] activate the cGAS-STING (cyclic GMP-AMP synthase linked to a stimulator of interferon genes) pathway, an intracellular immune system that promotes SASP secretion, especially in cancer cells. Activation of this pathway is linked to gene expression of inflammatory factors [14, 15]. CCFs are a type of cytoplasmic chromatin and often be observed in senescent cells caused by DNA damage, activation of oncogenes, or replication deficiency [15–17]. CCFs contain the heterochromatin markers H3K9me3 and H3K27me3 but lack the true chromatin marker H3K9ac. In addition, CCFs show a positive reaction for γH2AX, a DNA damage marker, but negative for p53 binding protein-1 (53BP1) [18]. However, the mechanism by which CCFs are formed in drug-induced senescent cells is far from explained. The nuclear pore complexes are embedded in the nuclear envelope, the double-membraned barrier that surrounds the nucleus, and is the only mediator that mediates the transport of macromolecules between the nucleus and the cytoplasm [19]. In senescent cells, the nuclear pore protein Tpr is essential for the development and maintenance of senescence-associated heterochromatin foci (SAHF) and is also involved in the heterochromatin exclusion zone within the nuclear pore complex (NPC) [20, 21]. Moreover, it has been reported that nucleoporin, such as Tpr, is increased in cancer cells after chemotherapy such as HDAC inhibitor treatment [22]. Therefore, we speculate that the nuclear pore complex may be involved in the drug-induced SASP and the formation of CCFs in cancer cells.

Histone deacetylase is a critical regulator of gene expression that catalyzes the removal of an acetyl group from the lysine residue of histones, leading to condensation of chromatin and transcriptional repression of their target genes, including tumor suppressor genes [23]. In several cancer types, such as lung and breast, overexpression of individual HDACs is correlated with significant reductions in disease-free and overall survival, and could predict poor patients' prognosis regardless of cancer type and disease status [24]. Hyperacetylation of histones and non-histone proteins by HDAC inhibitors revealed various anticancer effects, such as apoptosis and cell cycle arrest in cancer cells [24, 25]. SAHA (suberoylanilide hydroxamic acid) is the first HDAC inhibitor approved by the FDA to treat refractory cutaneous T-cell lymphoma and has shown anticancer activity in several solid tumors including hematological malignancies [26, 27], NSCLC [28], and SCLC [29]. SAHA also induces senescence in various cancer cells [30], and it is correlated to the secretion of SASP during senescence [31]. However, the mechanism by which SAHA induces SASP remains unclear, and the relationship between SAHA and CCF has not yet been reported either.

EZH2 (enhanced zeste homolog 2), a catalytic subunit of PRC2 (polycomb repressive complex 2), is a methyltransferase that mediates trimethylation of lysine 27 on histone H3, suppressing the expression of PRC2 target genes [32]. Many studies have shown that overexpression of EZH2 in tumor tissues is closely related to tumor malignancy, poor therapeutic effect, and worse survival [33–35]. The expression level of EZH2 is high in lung cancer, particularly in SCLC, which is associated with poor therapeutic effects, making it a mark for lung cancer treatment [33, 36, 37]. EPZ-6438, which directly targets PRC2, is an oral drug already approved by the FDA to treat epithelioid sarcoma and follicular lymphoma [38, 39]. In particular, it has already been suggested that EZH2 promotes the formation of CCFs in breast cancer cells and promotes breast cancer metastasis through the activation of the cGAS-STING pathway by CCFs [40].

In the present study, we demonstrated that SAHA induced the secretion of SASP, which was inhibited by EZH2 inhibitors in SCLC cell lines. We showed that SAHA promoted CCF production and activated the cGAS-STING pathway through CCFs. We further revealed that SAHA-induced CCFs was the result of an increase in nuclear pore density and inhibited by EZH2 inhibitors. Moreover, we found that combined treatment of cells with SAHA and EZH2 inhibitors induced cellular senescence via the suppression of CCF production, resulting in the inhibition of SASP secretion and the enhancement of the cancer-suppressing effect of SAHA in SCLC cells. These findings may help to develop a new therapeutic strategy for SCLC.

## Result

### SAHA induces SASP in SCLC

SAHA, an FDA-approved HDAC inhibitor, was used to treat various types of cancers [26–29] and induced senescence in several cancer cells [30]. To determine whether SAHA can induce senescence in SCLC cells, cells were treated with SAHA.

**Fig. 1:**
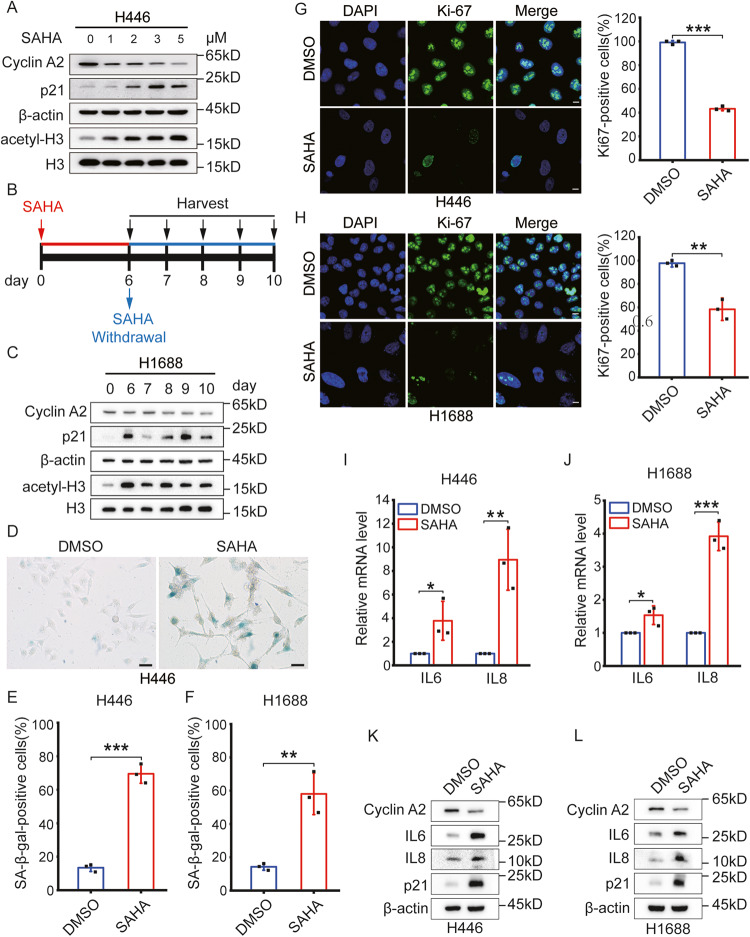
SAHA induces senescence and SASP in SCLC cell lines. **A** Western blotting was used to evaluate the expressions of senescence-associated proteins in H446 cells treated with various concentrations of SAHA for six days. **B** Schematic illustration of experimental design in **C**. **C** H1688 cells exposed to 3 μM SAHA for 6 days, after SAHA withdrawal, and determined the time of occurring cell cycle arrest using western blotting. **D** SA-β-gal images of H446 cells treated with DMSO or 3 μM SAHA for 6 days. Scale bars = 50 µm. **E** Shown is the percentage of positive cells over 100 cells. *n* = 3 independent experiments. **F** H1688 cells were treated with DMSO or 3 μM SAHA for 6 days, SA-β-gal activity presents the percentage of positive cells over 100 cells at two days after SAHA withdrawal. *n* = 3 independent experiments. **G**, **H** Immunofluorescent analysis of SCLC cell lines stained with Ki-67 to determine senescence. **G** H446 cells were treated with DMSO or 3 μM SAHA and analyzed six days later. *n* = 3 independent experiments. **H** H1688 cells were treated with DMSO or 3 μM SAHA for 6 days and analyzed 2 days after SAHA withdrawal. Scale bars = 10 µm. Shown is the percentage of positive cells over 100 cells. *n* = 3 independent experiments. **I** RT-PCR determined the mRNA levels of IL6 and IL8 in H446 cells after treatment of DMSO or 3 μM SAHA for 6 days. *n* = 3 independent experiments. **J** The mRNA levels of IL6 and IL8 in H1688 cells treated as above were determined using RT-PCR. mRNA levels were quantified relative to β-actin (control) mRNA. All experiments were repeated three times and presented as Mean ± SD. *n* = 3 independent experiments. **K** The protein levels of IL6 and IL8 were analyzed in H446 cells treated with DMSO or 3 μM SAHA for 6 days by western blotting. **L** H1688 cells also were treated as above, and IL6 and IL8 protein levels were analyzed using western blotting. SAHA suberoylanilide hydroxamic acid. *P < 0.05;**P < 0.01; ***P < 0.001 compared with the corresponding control.

We exposed the SCLC cell lines to different doses of SAHA, and identified concentrations that impeded cell proliferation in SCLC H446 and H1688 cells. We observed that SAHA at 3 μM decreased the expression level of Cyclin A2 in H446 cells (Figs. S1A and 1A). Reduced levels of Cyclin A2 and elevated levels of p21 after 6 days of SAHA treatment in H446 cells confirmed senescence, as shown in Western analysis (Fig. S1B). However, SAHA did not cause the reduction of Cyclin A2 in another SCLC H1688 cell line (Fig. S1C, D), despite that the treatment of SAHA increased the expression of p21, one of the main drivers of cell cycle arrest in senescence [31, 41]. We treated H1688 cells with 3 μM of SAHA for 6 days and determined the time of occurring the reduction of Cyclin A2 after SAHA withdrawal (Fig. 1B). In H1688, we also confirmed the reduced levels of Cyclin A2 and raised levels of p21 2 days after SAHA withdrawal (Fig. 1C). In SAHA-treated SCLC cells, senescence was further confirmed by SA-β-gal activity and Ki-67 expression. The rise in SA-β-gal activity and the decrease in Ki-67 immunofluorescence expression were identified in SAHA-treated SCLC cells (Fig. 1D–H).

SASP [8] promotes tumorigenesis and tumor progression, triggers adverse effects on cancer treatment [12], and is also a marker for judging the senescence state [7]. To determine whether SAHA induces SASP, we measured the secretion of IL6 and IL8, which are major SASP factors. As confirmed by the elevated levels of mRNAs encoding IL6 and IL8 using qPCR (Fig. 1I, J), and the high expression of IL6 and IL8 proteins using western analyses (Fig. 1K, L), SAHA promoted the secretion of SASP in SCLC cells. These data indicate that SAHA not only induces senescence, but also promotes the secretion of SASP in SCLC cells, at the same time.

### SAHA promotes the secretion of SASP by inducing CCF in SCLC

**Fig. 2:**
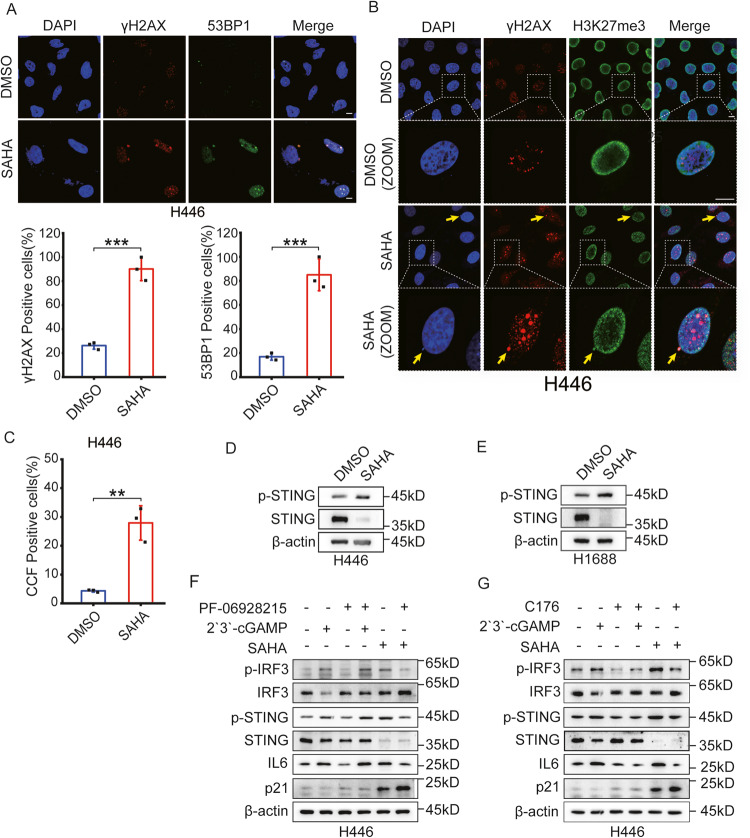
CCF associated with SAHA-induced SASP in SCLC cell lines. **A** Immunofluorescent images stained with γΗ2ΑΧ and 53BP1 were used to evaluate the DDR in H446 cells after treatment of DMSO or 3 μM SAHA for 6 days. Results were evaluated by the average values of five different fields with over 100 cells. Scale bars = 10 µm. *n* = 3 independent experiments. ***P < 0.001 compared with the corresponding control. **B** Immunofluorescent images stained with γΗ2ΑΧ and H3K27me3 were used to calculate the proportion of CCF in H446 cells after treatment of DMSO or 3 μM SAHA for 6 days. CCF was indicated by arrows. Scale bars = 10 µm. **C** The proportion of CCF was assessed as the average of five different fields with over 100 cells in H446 cells. Error bars indicate Mean ± SD, Student’s *t*-test. *n* = 3 independent experiments. **P < 0.01 compared with the corresponding control. **D** The protein levels of STING and p-STING were presented in H446 cells treated with DMSO or 3 μM SAHA by western blotting 6 days later. **E** H1688 cells determined the protein levels of STING and p-STING by western blotting. H1688 cells were treated with DMSO or 3 μM SAHA for 6 days and used for western blotting at SAHA withdrawal two days later. **F** Western blotting indicated the expression levels of cGAS-STING pathway-related proteins in H446 cells after co-treatment of 3 μM SAHA and 10 μM PF06928215. SAHA was treated for 6 days, and PF06928215 treatment time was 24 h. For positive control of the cGAS-STING activation, H446 cells were treated with 5 µM cGAMP for 4 h. **G** DMSO or 3 μM SAHA-treated H446 cells for 6 days were treated with 1 μM STING inhibitor C176 for 48 h and measured by western blotting.

Senescence-related SASP factors occur with persistent DNA damage signaling, especially DNA double-strand breaks (DSBs). DNA damage markers phosphorylated H2AX (γH2AX) and 53BP1 rapidly localize to DSBs, producing distinctive foci [42]. We stained SCLC cells after treating SAHA with γH2AX and 53BP1 antibodies to determine whether SAHA induces DNA damage response (DDR) in SCLC cell lines. Consistent with the previous study [18], SAHA-induced senescent cancer cells also included elevated expression levels of γΗ2ΑΧ and 53BP1, as measured by immunofluorescence assay (Figs. 2A and S2A). In multiple models of senescence induced by DNA damage, replication inhibition, and the oncogene HRasV12, there were CCFs, stained positive with DAPI (4',6-diamidino-2-phenylindole), γH2AX, and H3K27me3 [16, 18]. We, therefore, examined the localization of γH2AX and H3K27me3 and observed that CCF existed in SCLC cell lines. These results showed that SAHA produced DDR and increased CCF with positive for γH2AX and H3K27me3 in SCLC cell lines, at the same time (Figs. 2B, C and S2B). This is in agreement with the previous finding that the CCF is related to DDR [16]. Our results thus show that SAHA induces DDR and subsequently causes the accumulation of CCF in SCLC cell lines.

CCF, the outcome of pathogen infection and senescence, is recognized by cGAS, and activation of cGAS promotes the production of SASP factors through activating STING [13, 15]. In SCLC cell lines, STING expression is often markedly lessened or abolished, harming the activity of the STING pathway [43]. However, a study has reported that STING is expressed in some SCLC cell lines [44]. Furthermore, there have been no reports about the expression of STING in H446 and H1688 cells. We thus examined the expression of STING in H446 and H1688 cells. The result showed that the level of STING in H446 and H1688 cells were not low (Fig. S2C). Next, we assessed whether SAHA could activate the cGAS-STING pathway by detecting the expression of p-STING and STING proteins using western blotting. As shown in Fig. 2D, E, increased p-STING after treatment of SAHA was observed. This result shows that SAHA can promote the production of SASP factors through the cGAS-STING pathway. In particular, when C176, a specific inhibitor of STING, or cGAS inhibitor PF06928215 was used with SAHA, the expression level of IL6 protein as well as p-IRF3 and p-STING all decreased in SCLC cell lines, but the expression state of p21 protein, which reflects the senescent state, did not change with the secretion state of SASP (Figs. 2F, G and S2D, E). Altogether, these results indicated that SAHA promoted the secretion of SASP through the cGAS-STING pathway by inducing CCF in H446 cells.

### CCF is related to nuclear pore density in SAHA-treated SCLC cells

**Fig. 3:**
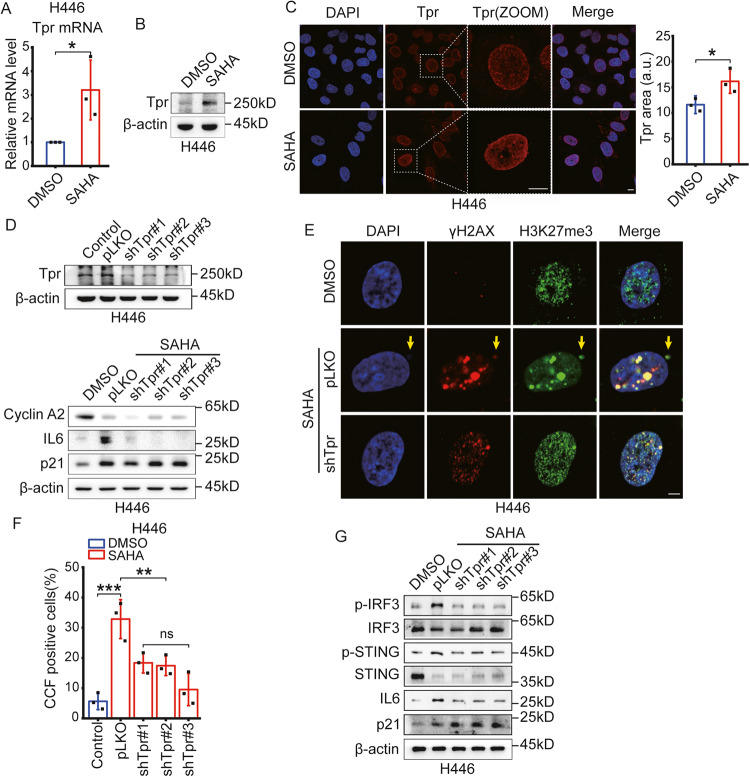
The nuclear pore density increases and is related to CCF after treatment of SAHA in SCLC cells. **A** The relative mRNA level of Tpr in H446 cells after treatment of DMSO or 3 μM SAHA for 6 days was evaluated using RT-PCR. Error bars represent Mean ± SD, and the results are the average of three different experiments. mRNA levels were quantified relative to β**-**actin (control) mRNA. **B** Western blotting showed the expression levels of Tpr in H446 cells treated with DMSO or 3 μM SAHA for 6 days. **C** H446 cells were immunofluorescent stained with Tpr after treatment of DMSO or 3 μM SAHA for 6 days, evaluating the change of nucleoporin Tpr area. Scale bars = 10 µm. *n* = 3 independent experiments. **D** H446 cells transfected with a shTpr plasmid were treated with DMSO or 3 μM SAHA for 6 days and measured by western blotting. **E** Immunofluorescent images showing the incidence of CCF in H446 cells inhibited Tpr protein using interfering mRNA. Scale bars = 5 µm. **F** The proportion of CCF was assessed as the average of five different fields over 100 cells after treatment of DMSO or 3 μM SAHA for 6 days in H446 cells transfected with a shTpr plasmid. Error bars indicate Mean ± SD. *n* = 3 independent experiments. **G** The protein levels of the cGAS-STING pathway and senescence-associated proteins in H446 cells transfected with a shTpr plasmid were assessed by western blotting after treatment of DMSO or 3 μM SAHA for 6 days. *ns*, no significant; *P < 0.05; **P < 0.01; ***P < 0.001 compared with the corresponding control.

Between cell nuclei and cytoplasm, biomolecules are not apparent in how they move through NPC [45], but various studies have found to migrate the mRNA and proteins through NPC [46, 47]. In addition, it has been reported that nuclear pore density increases during senescent cells, which is related to the secretion of SASP [20]. Therefore, we speculated that the emergence of CCF, a crucial factor for the secretion of SASP, would be related to NPC. In particular, Tpr, one of the nuclear pore complex proteins, is essential in the heterochromatin exclusion region [21]. First, we analyzed the expression of Tpr after treatment of SAHA in SCLC cell lines to assess the relationship between SAHA and NPC. We found that mRNA and protein levels of Tpr significantly increased after SAHA treatment in SCLC cell lines (Figs. 3A, B and S3A, B). Also, as seen through immunofluorescence analysis by labeled Tpr, nuclear pore density increased in SCLC cell lines after SAHA treatment (Figs. 3C and S3C).

Next, to evaluate whether Tpr affects SASP induced by SAHA, we inhibited Tpr mRNA transcription using Tpr-interfering mRNA in SCLC cell lines. After suppressed Tpr expression, the IL6 expression level significantly decreased in SAHA-treated SCLC cell lines (Figs. 3D and S3D). In addition, there was no change in the expression status of senescent markers such as Cyclin A2 and p21 in SAHA-treated cancer cells after Tpr expression inhibition. Therefore, we speculated that Tpr was only related to the secretion of SASP in SAHA-induced cellular senescence. As shown in the above experiments, the secretion of SASP in SAHA-treated SCLC cell lines is closely related to the emergence of CCF. Based on these results, we speculated that the emergence of CCF in SAHA-treated SCLC cell lines may also be linked to NPC. To evaluate the relationship between Tpr and CCF, we treated SAHA in SCLC cells that suppressed the expression of Tpr, and measured the incidence of CCF using immunofluorescence analysis for γH2AX and H3K27me3. As expected, the incidence of CCF significantly decreased after Tpr expression inhibition (Figs. 3E, F and S3E), then the cGAS-STING pathway-related proteins were also inhibited, as seen in the expression levels of p-IRF3 and p-STING (Figs. 3G and S3F). These results show that the emergence of CCF is closely linked to NPC, and SAHA increases the NPC density in SCLC cell lines, increasing the amount of CCF, and thus promoting the production of SASP.

### Inhibition of EZH2 suppresses SAHA-induced SASP in SCLC

**Fig. 4:**
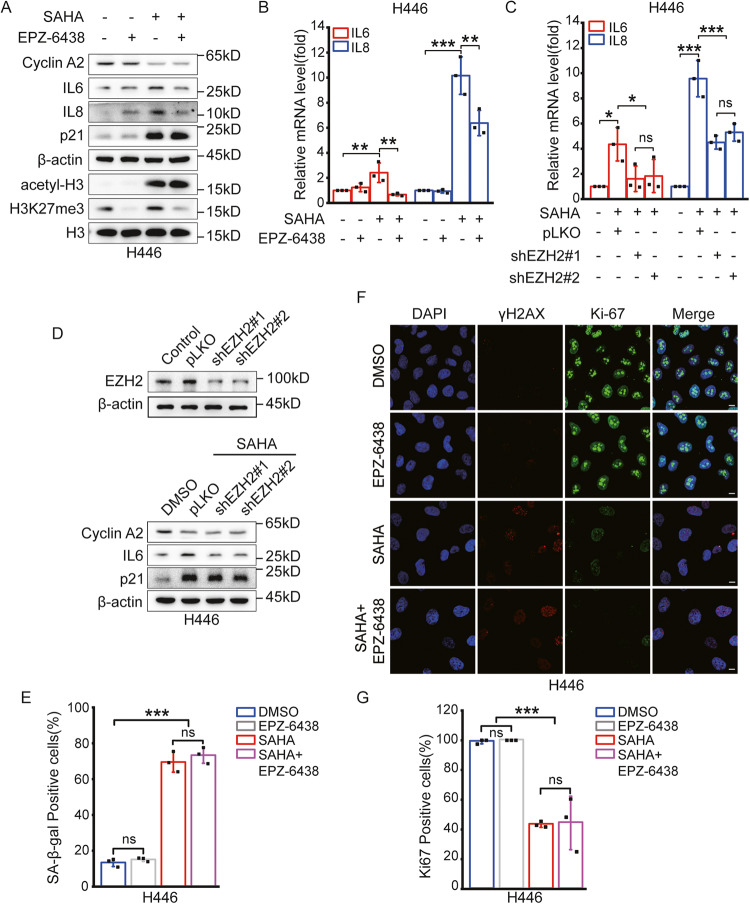
EZH2 inhibitor EPZ-6438 suppresses SAHA-induced SASP in SCLC cells. **A** Western blotting was used to assess the expression levels of senescence-associated proteins in H446 cells treated with DMSO or 3 μM SAHA and 0.5 μM EZP-6438 for 6 days. **B** The mRNA levels of IL6 and IL8 in H446 cells after treatment of DMSO or 3 μM SAHA and 0.5 μM EZP-6438 for 6 days were detected using RT-PCR. mRNA levels were quantified relative to β-actin (control) mRNA and presented as Mean ± SD of three independent experiments. **C** A shEZH2 plasmid was transfected into H446 cells. RT-PCR was used to examine the mRNA levels of IL6 and IL8 after treatment of DMSO or 3 μM SAHA for 6 days in H446 cells transfected with a shEZH2 plasmid. *n* = 3 independent experiments. **D** The expression levels of senescence-associated proteins in H446 cells transfected with shEZH2 plasmid was assessed after treatment of DMSO or 3 μM SAHA for 6 days by western blotting. **E** H446 cells were treated with DMSO or 3 μM SAHA and 0.5 μM EZP-6438 for 6 days and detected SA-β-gal activity. SA-β-gal activity in H446 are the percentage of the positive cells relative to cells detected, repeated three times independently. **F** Immunofluorescent images were used to assess the senescent state of H446 cells treated with DMSO or 3 μM SAHA and 0.5 μM EZP-6438 for 6 days. Scale bars = 10 µm. **G** Results are the percentage of positive cells over 100 cells in H446 cells. *n* = 3 independent experiments. ns no significant; *P < 0.05; **P < 0.01; ***P < 0.001 compared with the corresponding control.

Studies have found that CCF is positive for H3K27me3 [16]. EZH2, a methyltransferase mediating H3K27me3 modification in the cell nucleus, is generally highly expressed in lung cancer [33, 36]. We recently demonstrated that EZH2 is closely linked to the formation of CCF and metastasis in breast cancer [40]. Therefore, we speculated that SASP and CCF induced by SAHA may be related to EZH2, too. In particular, EPZ-6438, a specific inhibitor of EZH2, is an oral drug approved by the FDA [38, 39]. We first compared the EZH2 level in various SCLC cell lines and observed that the EZH2 level in various SCLC cell lines was similar (Fig. S4A). Next, we determined the appropriate dose by combining SAHA with different concentrations of EPZ-6438. We found that the reduction of IL6 in SAHA-induced senescent cells depended on the concentration of EPZ-6438, as shown in Western analysis (Fig. S4B, C). The combined treatment of SAHA and EPZ-6438 caused no significant change in Cyclin A2 and p21 expression, but in IL6 protein expression level. Based on these results, we chose the dose 0.5 μM of EPZ-6438, which, in combination with SAHA, maintained SAHA-induced senescence, and especially inhibited the expression of IL6, a factor of SASP. In the selected co-treatment, senescence induced by SAHA was maintained, and the expression levels of IL6 and IL8 was significantly reduced, as shown by western blotting (Figs. 4A and S4D). A study reported that the suppression of EZH2 increased the expression of p21 and induced senescent phenotype [48]. But the senescence-related proteins, such as Cyclin A2 and p21, did not change when SCLC cells were exposed to EPZ-6438 alone, suggesting that SCLC cells were not sensitive to EPZ-6438 in inducing senescence. Next, we assessed the mRNA level of IL6 and IL8 using qPCR analysis. The results show that it also reduces the mRNA levels of IL6 and IL8 after the combined treatment of SAHA and EPZ-6438 (Figs. 4B and S4E). In addition, to further confirm the effect of EPZ-6438, we inhibited EZH2 mRNA transcription using the interfering mRNA of EZH2 and evaluated the expression of senescence-related proteins in SCLC cell lines treated with SAHA. The expression levels of IL6 and IL8 were remarkably repressed in SCLC cells that suppressed EZH2 transcription using interfering mRNA (Figs. 4C, D and S4F, G), suggesting that EZH2 is closely related to the secretion of SASP in SAHA-induced senescent SCLC cells.

Next, we determined the changes in the senescent state after co-treatment with SAHA and EPZ-6438. In cancer treatment, most of the chemotherapies are accompanied by senescence [9–11], and senescence occurring during this process is regarded as an effective mechanism to prevent cancer growth [49, 50]. Therefore, we examined the senescent state of cancer cells after the combined treatment of the two drugs. After the combined treatment of the two drugs, the senescence-associated β-galactosidase activity was not different from when SAHA was used alone (Figs. 4E and S4H), and the expression of Ki-67 was still suppressed with SAHA treatment alone (Figs. 4F, G and S4I, S4J). Therefore, our results show that the combined treatment of SAHA and EZP-6438 suppressed the secretion of SASP derived by SAHA, but it does not change the senescent state derived by SAHA.

### EZH2 promotes the formation of CCF under SAHA treatment

**Fig. 5:**
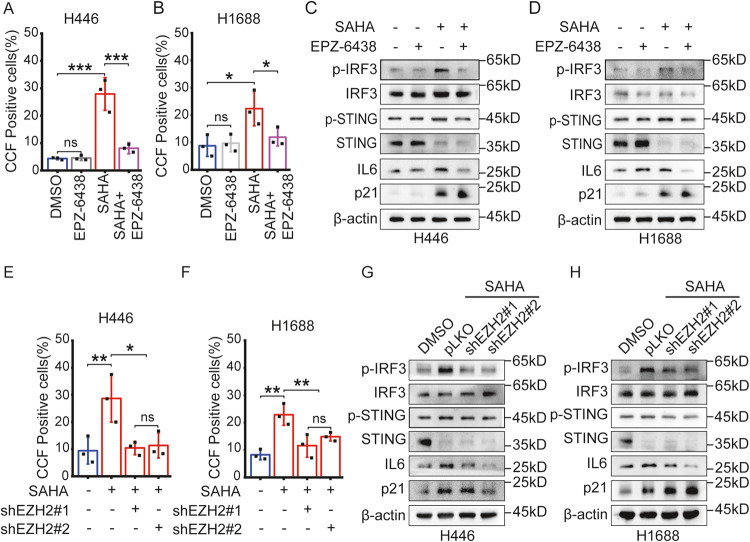
EZH2 inhibitor EPZ-6438 suppresses SAHA-induced SASP by decreasing CCF in SCLC cells. **A**, **B** H446 cells (**A**) and H1688 cells (**B**) were immunofluorescent stained with γΗ2ΑΧ and H3K27me3 to evaluate the incidence of CCF after treatment of DMSO or 3 μM SAHA and 0.5 μM EZP-6438, co-treatment of 3 μM SAHA and 0.5 μM EZP-6438, for 6 days, respectively. In H1688 cells, 3 μM SAHA was treated in a separate SAHA treatment area for 6 days and immunofluorescence was performed after 2 days. The incidence of CCF was presented as Mean ± SD of five different fields over 100 cells. *n* = 3 independent experiments. **C**, **D** Western blotting was used to assess the expression levels of cGAS-STING pathway and senescence-associated proteins in H446 (**C**) and H1688 (**D**) cells treated for 6 days with DMSO or 3 μM SAHA and 0.5 μM EZP-6438, co-treatment of 3 μM SAHA and 0.5 μM EZP-6438, respectively. According to the above method, SAHA alone treatment region was performed in H1688 cells. **E**, **F** The incidence of CCF in H446 (**E**) and H1688 (**F**) cells transfected with a shEZH2 plasmid were evaluated using immunofluorescence after treatment of 3 μM SAHA for 6 days. The values represent the Mean ± SD of five different fields over 100 cells. *n* = 3 independent experiments. **G**, **H** The expression levels of the cGAS-STING pathway and senescence-associated proteins in H446 (**G**) and H1688 (**H**) cells transfected with a shEZH2 plasmid were evaluated using western blotting after treatment of 3 μM SAHA for six days. ns no significant; *P < 0.05; **P < 0.01; ***P < 0.001 compared with the corresponding control.

As shown from the above results, since the combined treatment of SAHA and EZH2 inhibitors inhibited SASP, we speculated which might be related to CCF. First, we observed changes in CCF after co-treatment with SAHA and EPZ-6438. As seen by immunofluorescence analysis, the incidence of CCF significantly reduced after co-treatment with SAHA and EPZ-6438 (Fig. 5A, B). In addition, we determined whether the cGAS-STING pathway, the downstream pathway of CCF, was activated after co-treatment with SAHA and EPZ-6438 using Western analysis. As expected, western blotting analysis confirmed that the expression levels of p-IRF3, p-STING, and IL6 were reduced after co-treatment with SAHA and EPZ-6438 (Fig. 5C, D), indicating that it suppressed the secretion of SASP by inhibiting the cGAS-STING pathway in SCLC cells. Therefore, these results show that inhibition of H3K27me3, one of the CCF markers, inhibits CCF, a crucial mediator in the secretion of SASP by SAHA in SCLC cells, thereby inhibiting the secretion of SASP through suppression of the cGAS-STING pathway. In SCLC cells with EZH2 expression suppressed by EZH2 mRNA interference, the incidence of CCF was also significantly reduced after SAHA treatment (Fig. 5E, F), and the expression of cGAS-STING pathway-related proteins was also inhibited (Fig. 5G, H). Therefore, these results show that inhibition of H3K27me3, one of the CCF markers, inhibits CCF, thereby inhibiting the secretion of SASP via the suppression of the cGAS-STING pathway.

### Inhibition of SASP alleviates the proliferation of SAHA-treated SCLC cells

**Fig. 6:**
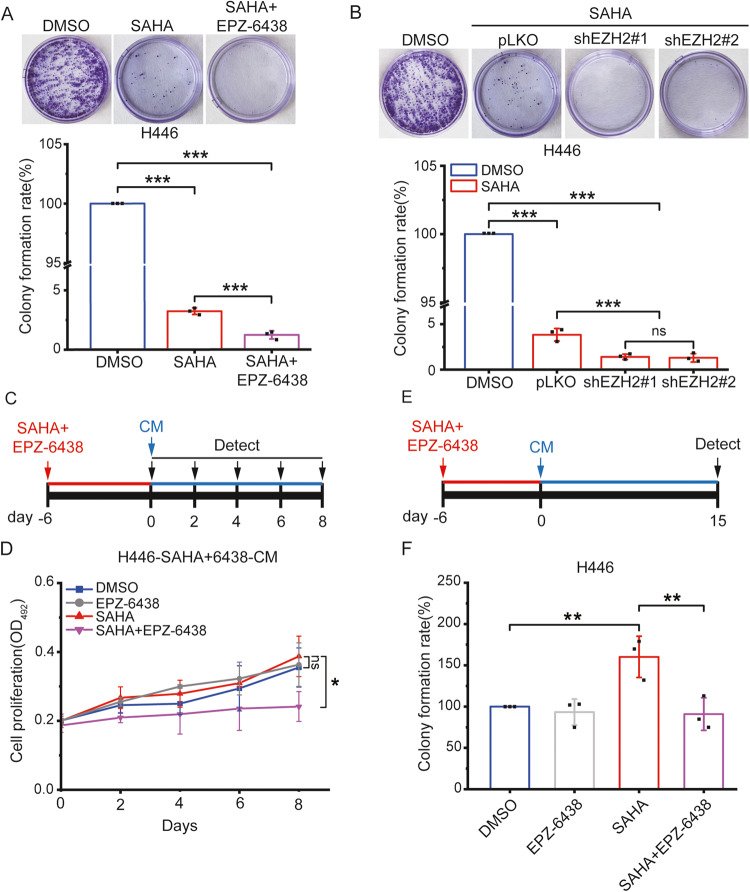
The inhibition of SASP potentiates the antiproliferative effect of SAHA in H446 cells. **A** The colony formation rate was assessed as the percentage of colonies formed in the presence of a normal medium after treatment of 3 μM SAHA, the combination of 3 μM SAHA and 0.5 μM EZP-6438 for 6 days in H446 cells. The colony formation assay was performed ten days after drug withdrawal. *n* = 3 independent. **B** H446 cells transfected with a shEZH2 plasmid were treated with 3 μM SAHA for 6 days and the colony formation rate was detected after incubating in a normal medium. The colony formation assay was conducted on the same day. *n* = 3 independent. **C** Schematic summary of the experimental design of SASP-induced proliferative experiment in **D**. **D** H446 cells were treated with the combination of 3 μM SAHA and 0.5 μM EZP-6438 for 6 days and subsequently incubated for eight days in the presence of CM, the cell proliferation was examined using an MTT assay. *n* = 3 independent **E** Schematic summary of the experimental design to evaluate SASP-induced colony formation rate in **F**. **F** H446 cells were treated with the combination of 3 μM SAHA and 0.5 μM EZP-6438 for 6 days and subsequently incubated in the presence of CM, the colony formation rate was analyzed 15 days later. The values of all experiments represent the Mean ± SD of three independent experiments. ns no significant; *P < 0.05; **P < 0.01; ***P < 0.001 compared with the corresponding control.

The SASP factor weakens the anticancer action of the drug by promoting the proliferation of cancer cells [12]. As seen from the above experimental results, SAHA-treated cancer cells secreted SASP, and when combined SAHA with EZP-6438, the secretion of SASP factor was suppressed in SCLC cells. We first examined the colony formation rate after treatment of SAHA and EPZ-6438 for 6 days to evaluate the antiproliferative effects of co-treatment in SCLC cell lines. As expected, the combination of SAHA and EPZ-6438 significantly inhibited the ability of colony formation compared with SAHA alone (Figs. 6A and S5A). In SCLC cells with EZH2 expression suppressed by a shEZH2 plasmid, the ability of colony formation was inhibited compared with cells transfected with a pLKO plasmid (Figs. 6B and S5B). These results indicated that the co-treatment of SAHA and EZP-6438 might enhance the SAHA antiproliferative effect in SCLC cells.

Next, we evaluated the effect of SASP inhibition in SCLC cells. To assess the influence of SASP on the proliferation of cancer cells, we examined the proliferation of cancer cells in CM. As evaluated from the results of the MTT experiment, there were no significant differences in the proliferative rate of SCLC cells in each CM, respectively (Fig. S5C, D). This result shows that SASP does not affect the proliferation of SCLC cells. This may be because the proliferation of cancer cells is already very fast [2]. We then tested whether SASP affected the antiproliferative effect of co-treatment with SAHA and EPZ-6438. SCLC cells were incubated sequentially in each CM after co-treatment of SAHA and EPZ-6438 for 6 days (Fig. 6C). We confirmed that the antiproliferative effect in the presence of SASP was significantly lower than in the medium without SASP (Figs. 6D and S5E). Consistent with the results of previous studies [12], SASP also promoted the proliferation of SCLC cells treated with SAHA and EPZ-6438. In addition, we examined whether SASP stimulated colony formation in SCLC cells treated with SAHA and EPZ-6438. As the above experimental method, SCLC cells that were treated with SAHA and EPZ-6438 for six days were incubated sequentially in CM, respectively, and the rate of colony formation was analyzed fifteen days later (Fig. 6E). The CM obtained from each SCLC cell line treated with SAHA and EPZ-6438 also significantly inhibited the capability of colony formation compared to that obtained from SCLC cells treated with SAHA (Figs. 6F and S5F). These results show that SASP promotes the proliferation of SCLC cells, and the suppression of SASP might alleviate the hyperplasia of SCLC cells treated with SAHA and EPZ-6438.

Our finding that the combined treatment of SAHA and EPZ-6438 promotes senescence and suppresses the secretion of SASP in SCLC cells predicts that the co-treatment of both inhibitors could abolish the progress of the tumor effectively under various circumstances. To assess this probability, we performed bio-information analyses of gene expression datasets. Kaplan–Meier analysis showed that overexpression of both inhibitors targeting genes was associated with worse survival of lung cancer patients (Fig. S6A, B). Therefore, there is evidence that SAHA combined with EPZ-6438 can inhibit the progression of SCLC tumors.

## Discussion

In this study, we found that SAHA-induced senescence in SCLC cells was accompanied by SASP, and the production of SASP was related to the formation of CCF in SCLC cells treated with SAHA. SAHA promotes the formation of CCF and activates the cGAS-STING signal pathway, thus producing SASP. In particular, EZH2 assists the formation of CCF in SCLC cells treated with SAHA and participates in the production of SASP. Therefore, the combined treatment of SAHA and EZH2 inhibitors can induce the senescence of SCLC cells, and inhibit the secretion of SASP, thus inhibiting the proliferation of SCLC cells treated with SAHA.

Cells can undergo senescence in response to DNA damage, oxidative stress, telomere shortening, and oncogenic stimuli [14]. It has been reported that anticancer drugs induce senescence of cancer cells during the treatment process and that SAHA also induces senescence phenotypes in various cancer cells. For example, SAHA was shown to induce senescence in colon cancer cells HCT116 (at 2 μΜ for 24 h), human leukemia cell lines MOLM-7, HL-60, and JURL-MK1 (0.5–10 μM for 72 h), urothelial carcinoma (2.5 μM for 48 h), glioblastoma stem cells (2.5 μM for 7 days) and adenoid cystic carcinoma primary cells (1 or 1.4 μM for 120 h) [30]. Our results showed that SAHA induced senescence in SCLC cell line H446. In particular, at the concentration of 3 μΜ of SAHA, the senescent status is evident in H446 cells, and the senescence was induced after 6 days. But in H1688 cells, the same concentration did not results in any change in the cycle-related protein Cyclin A2 during the same period (Fig. S1D). However, like in H446 cells, the expression of the senescence-associated protein p21 increased after SAHA treatment and underwent senescence two days after SAHA withdrawal (Fig. 1C). According to the expression of neuroendocrine markers L-DOPA decarboxylase and neuro-specific enolase, SCLC cells are roughly characterized by neuroendocrine SCLC and non-neuroendocrine SCLC. Neuroendocrine SCLC cells are divided into SCLC-A and SCLC-N subtypes according to the expression characteristics of two lineage transcription factors, achaete-scute homologoue 1 (ASCL1) and neurogenic differentiation factor 1 (NeuroD1). In addition, non-neuroendocrine SCLC cells are classified into SCLC-Y and SCLC-P subtypes according to the expression of yes-associated protein-1 (YAP1) and POU class 2 homeobox 3 (POU2F3) [51]. H446 cells are a type of SCLC-N cells [51], and the characteristics of the H1688 cells derived from liver metastasis taken from a patient prior to therapy are not yet clear. This result suggests that the response to drug treatment is cell-type specific. Although the period of SAHA-inducing senescence is different in two kinds of SCLC cells, we confirmed that the generation of CCF and SASP through activating the cGAS-STING signaling pathway by CCF is the same under the senescent state, which is consistent with the previous reports [14, 16, 18]. SASP can promote the proliferation of tumor cells in different circumstances [30]and reverse the effects of chemotherapy [50]. Therefore, inducing senescence and inhibiting SASP in chemotherapy is an important issue.

CCF is a chromatin fragment that appears in the cytoplasm and originates from the nucleus of senescent cells and pathogenic infections [13]. CCFs have emerged as a form of nuclear budding in senescent cells, unlike micronuclei occurring from lagging chromosomes or acentric chromosome fragments that do not integrate into daughter nuclei [18, 52]. In addition, studies have reported that nuclear pore density increases in senescent cells [20], and nucleoporins such as TPR accumulate after treatment of HDAC inhibitors in the nucleus of cancer cells [22], resulting in the secretion of SASP. NPC is the main transport channel between the nuclear and the cytoplasm, and most nuclear-cytoplasmic exchange occurs through the NPC [19]. According to the data mentioned above, we reckoned that there must be a connection between NPC and CCF. First, we found that the expression of Tpr was upregulated in SCLC cells after SAHA treatment, and the number of nuclear pores was also increased, indicating that SAHA was closely related to the upregulation of nuclear pore density in SCLC cells. Then, we found that the expression of IL6 in SCLC cells with Tpr knockdown was significantly reduced after SAHA treatment. We also showed that after SAHA treatment, the proportion of CCF in SCLC cells with Tpr knockdown significantly decreased compared with the SAHA alone treatment group, the cGAS-STING signal pathway-associated protein p-IRF3 and p-STING, and SASP-associated protein IL6 were significantly down-regulated. These indicated that the increase in nuclear pore density promoted the formation of CCF in SCLC cells. We showed that the knockdown of Tpr inhibited the formation of CCF. It has been reported that Tpr is necessary for the occurrence and maintenance of SAHF and participates in the heterochromatin exclusion region in the nuclear pore complex [20, 21]. Therefore, the formation of CCF may be related to these functions of Tpr. Charlene Boumendil [20] et al. found that the knockdown of Tpr in senescent cells did not significantly change nuclear pore density. Given the increase of nuclear pore density in SCLC cells treated with SAHA, the formation of CCF could be related to the nuclear pore complex, especially Tpr, because Tpr participates in the heterochromatin exclusion region in the nuclear pore complex. The combination of SAHA and EZH2 inhibitor EPZ-6438 did not affect the expression of Tpr in SCLC cells. In SCLC cells, the mRNA level of Tpr was upregulated after SAHA treatment, but there was no significant change in the mRNA level of Tpr after a combined treatment of SAHA with EPZ-6438 compared with SAHA alone treatment (not shown), indicating that inhibition of EZH2 did not affect the increased expression of Tpr in SCLC cells treated with SAHA. However, how CCF moves to the cytoplasm through NPC needs further investigation.

Some studies have reported that EZH2 is expressed highly in most SCLC cells, an oncogenic factor associated with cancer development [53]. In addition, it has been reported that the overexpression of EZH2 promotes ASCL1 expression by repressing the transforming growth factor-β (TGFβ)–SMAD pathway through methylation, which in turn results in SCLC progression. Therefore, a specific EZH2 inhibitor was shown to effectively eliminate SCLC cells by TGF-β-mediated apoptosis [36]. In particular, a study already has found that the formation of CCF in breast cancer cells is correlated to the overexpression of EZH2 and promotes breast cancer metastasis through the secretion of inflammatory factors by the cGAS-STING pathway [40]. Thus, the EZH2-CCF-cGAS axis plays a crucial role in the secretion of inflammatory factors and tumor progression in breast cancer [40]. Therefore, we think that the induction of CCF by SAHA in SCLC cells may also be related to EZH2. As expected, after the combination treatment of SAHA and EPZ-6438, the incidence of CCF remarkably decreased, and the secretion of SASP was suppressed through the control of the cGAS-STING pathway. Moreover, the combination treatment of SAHA and EPZ-6438 only affected the secretion of SASP and did not affect the senescence of cancer cells caused by SAHA. This result shows that CCF is only implicated in the secretion of SASP independently of the senescence of cancer cells. Therefore, we show that EZH2 is fundamentally implicated in the stability of CCF and activation of cGAS in the cytoplasm, as previously found [40].

Conclusively, our results depict that SAHA produces CCF by controlling nuclear pore density in SCLC cells and subsequently activates the cGAS-STING pathway to promote the secretion of SASP. Our data also show that EZH2 is involved in the generation of CCF and secretion of SASP. The suppression of SASP may lead to side effects correlated to cancer treatments, and play a critical role in constructing therapeutic strategy, especially in SCLC, which easily acquires chemo-resistance and recurrence. Consequently, the promotion of senescence by SAHA and the suppression of SASP by EZH2 inhibition weakens the progression and relapse of tumors and may provide a therapeutic strategy to improve the treatment effects of SAHA in SCLC.

## Materials and methods

### Cell culture

Human small cell lung cancer (NCI-H446 and NCI-H1688), human cancer cell Hela, human breast cancer cell MCF-7, and human embryonic kidney 293 T (HEK293T) cells were purchased from the American Type Culture Collection (Manassas, VA, USA). Cells were amplified and frozen. New cells were revived from the frozen stocks every 1–2 months. Human small cell lung cancer cells were cultured in RPMI-1640 medium supplemented with 10% fetal bovine serum (FBS) (VivaCell, Shanghai, China), 100 units/mL penicillin and 100 mg/mL streptomycin (Sangon Biotech, Shanghai, China). Hela, MCF-7, and HEK293T cells were grown in Dulbecco`s modified Eagle medium (Sigma-Aldrich) supplemented with 10% FBS. To ensure that they were not contaminated with mycoplasma, cells were analyzed every 2 months using the Mycoplasma test kit (LT07-218, Lonza, Basel, Switzerland). Cell lines were cultured in a humidified incubator at 37 °C with 5% CO_2_. The H446 cells were incubated with the culture medium containing 3 μΜ of SAHA(Selleck, Catalog No.S1047) for 6 days and used for analysis. The H1688 cells were incubated with a culture medium containing 3 μΜ of SAHA for 6 days and analyzed 2 days later. For co-treatment of SAHA and EZH2 inhibitor, H446 and H1688 cells were incubated with the culture medium containing 3 μΜ of SAHA and 0.5 μΜ EPZ-6438(Selleck, Catalog No.S7128) and used for analysis. For positive control of the cGAS-STING activation, H446 and H1688 cells were incubated with the culture medium containing 5 μΜ 2'3'-cGAMP(APExBIO, Catalog No.B8362) for 4 h [40] and analyzed 24 h later. For treatment of STING inhibitor, 3 μM SAHA-treated H446 cells for 6 days were treated with 1 μM C176(Selleck, Catalog No.S6575) for 48 h [54] and measured, 3 μM SAHA-treated H1688 cells for 6 days were treated with 1 μM C176 for 48 h after SAHA withdrawal 2 days later and used for analysis. For treatment of cGAS inhibitor, 3 μM SAHA-treated H446 cells for 6 days were treated with 10 μM PF06928215 (MCE, Catalog No.HY-114182) for 24 h and measured, 3 μM SAHA-treated H1688 cells for 6 days were treated with 10 μM PF06928215 for 24 h after SAHA withdrawal 2 days later and used for analysis.

### siRNA transfection

Lentiviruses were generated in HEK293T cells and the lentivirus constructs were transfected into SCLC cells, according to the manufacturer’s instructions (Invitrogen). siRNA was chemically synthesized by JiMa Company (Shanghai, China) and their sequences are included in Supplement Table S1.

### SA-β-galactosidase staining

The staining solution was prepared using 20× KC [1.64 g K_3_Fe (CN)_6_, 2.1 g K_4_Fe (CN)_6_∗3H_2_O in 50 mL PBS] and 20× X-gal [40 mg/ml X-gal in *N*,*N*-dimethylformamide] diluted to 1× in pH 6.0 PBS/1 mM MgCl_2_. Cells were washed three times with PBS (pH 7.4) containing 0.5% glutaraldehyde, and fixed at room temperature for 15 min. And then cells were washed twice with PBS/MgCl_2_ (pH 6.0), and stained in the staining solution in a light-proof bag at 37 °C for 14–16 h. The result shows the percentage of positive cells over 100 cells. SA-β-gal positive cells are quantified by researchers who do not know the identity of the cells being analyzed.

### Immunofluorescence

Cells were washed twice with PBS, fixed with 4% paraformaldehyde solution for 10 min, and washed twice with PBS. Cells were permeabilized using permeabilization solution (0.5% Triton X-100 in PBS) for 10 min and washed twice with PBS. Cells were blocked in TBST (0.05% Tween 20 in TBS) containing 5% BSA for 1 h. Cells were incubated overnight in the corresponding primary antibody at 4 °C. After that cells were washed three times with TBST, 5 min each time, and then the corresponding secondary antibody was added, followed by incubation in a 37 °C incubator for 1 h. Cells were washed three times with TBST, 5 min each time, and then nuclei were counterstained with 500 nM DAPI (Sigma) at room temperature for 10 min. Images were obtained using a confocal microscope (ZEISS LSM880) equipped with ZEN imaging software. We calculated the area of γH2AX and 53BP1, Tpr within fluorescence images by Fiji ImageJ analysis software. The antibodies used were included in Supplementary Table S2. The cells are quantified by researchers who do not know the identity of the cells being analyzed.

### CCF determination and quantitative analysis

CCF markers γH2AX and H3K27me3 were used for immunofluorescence detection. The number of CCF in ≧100 cells was counted under an inverted microscope, and then the incidence of CCF was counted. The cells are quantified by researchers who do not know the identity of the cells being analyzed.

### Preparing of conditioned medium

SCLC cells (5 × 10^6^) were seeded in a 10 cm dish and incubated for 6 days in RPMI-1640 medium supplemented with 10% FBS and DMSO, 3 μM SAHA, 0.5 μM EPZ-6438, co-treatment of 3 μM SAHA and 0.5 μM EZP-6438, respectively. The medium was replaced with a fresh 10 mL RPMI-1640 medium supplemented with 10% FBS and incubated for 24 h. The medium was harvested, centrifuged at 1500 rpm for 5 min, and then filtered using a 0.2 μm membrane. Conditioned medium (CM) was mixed with RPMI-1640 medium supplemented with 40% FBS in a ratio of 3 to 1 to produce CM containing 10% FBS.

### Cell viability

Cells were seeded in a 96-well plate at a density of 2000 cells/well and incubated with a dose of SAHA diluted in RPMI-1640 medium supplemented with 10% FBS for 48 h. Cells treated with DMSO were used as control. About 20 μL of MTT solution (5 mg/mL MTT in PBS) was added to each well and incubated at 37 °C for 4 h. 100 μL dimethyl sulfoxide (DMSO) was added to each well and shaken on a shaker for 10 min at low speed to dissolve the crystal. The absorbance value of each well was measured at 492 nm.

### Cell proliferation

Cells were seeded in a 96-well plate at a density of 2000 cells/well and incubated with a dose of SAHA and EZP-6438 diluted in RPMI-1640 medium supplemented with 10% FBS for 8 days. The medium was replaced with the CM and incubated for 8 days. Cells treated with CM(DMSO) were used as control. The measuring of proliferation was carried out in the manner described above.

### Colony formation assay

Cells were seeded in a six-well plate at a density of 1 × 10^4^ cells/well and incubated with a dose of SAHA and EPZ-6438 diluted in RPMI-1640 medium supplemented with 10% FBS for 6 days. The medium was replaced with CM every three days and incubated for 15 days. The proportion of colony formation was counted as the percentage of colonies formed on the plate. The cells are quantified by researchers who do not know the identity of the cells being analyzed.

### Western blotting

The cell extracts were made by lysis of cells in 1×Laemmli sample buffer after washing in cold PBS. Protein lysates were resolved on SDS-PAGE and subsequently transferred to the PVDF membrane. The primary and secondary antibodies used for detection were reported in Supplementary Table S2.

### Reverse transcription, PCR, and real-time PCR analysis

Reverse transcription, PCR, and real-time PCR were carried out as described [40]. Total RNA was prepared from cells using an RNAiso Plus kit following the manufacturer’s instructions. The cDNA was made using the Reverse Transcription System. RT-PCR was performed using 2× Taq Master Mix. The relative expression of the targeting gene was measured using the 2^- ΔΔCt^ method, with β**-**actin mRNA as the internal control. The primers used for RT-PCR were listed in Supplementary Table S3.

### Statistical analyses

All data were compiled from the results of at least three independent experiments and represented as mean ± SD. The unpaired Student`s *t*-test (two-tailed) or two-way analysis of variance (ANOVA) was used to calculate the significance of differences between groups. Data were considered statistically significant at *P* < 0.05. Statistical parameters are reported in the figure legends. Statistical analysis was completed using Prism 8 (GraphPad Software, La Jolla, CA, USA).

### Supplementary information

Supplementary figure legends

Supplementary figure 1

Supplementary figure 2

Supplementary figure 3

Supplementary figure 4

Supplementary figure 5

Supplementary figure 6

Supplementary Table S1

Supplementary Table S2

Supplementary Table S3

Original Data File

## Data Availability

The data underlying this article are available on request from the corresponding author.
