# Supplementary material for: Inhibition of EZH2 alleviates SAHA-induced senescence-associated secretion phenotype in small cell lung cancer cells
Source: Cell Death Discov. 2023 Aug 5;9:289. doi: 10.1038/s41420-023-01591-y (PMC10404275; doi:10.1038/s41420-023-01591-y)
Supplement: Supplementary file 1 — Supplementary figure legends [file 41420_2023_1591_MOESM1_ESM.docx]

**EZH2 inhibitor EPZ-6438 alleviate SAHA-induced SASP by regulating CCF**

**in small cell lung cancer cells-Supplement**

**Supplement 1.** SAHA induces senescence differently in each other SCLC cell lines. (**A**) H446 cells and H1688 cells were exposed to different concentrations of SAHA for 72 h and were measured for viability using an MTT assay. The graph represented the absorbance of each sample as a percentage of control, results were represented as Mean±SD repeated three times. (**B-D**) The expressions of senescence-associated proteins as cyclin A2 and p21 were evaluated by western blotting in H446 cells treated with 3 μM SAHA for different days(**B**), in H1688 cells treated with various concentrations of SAHA for six days(**C**) and H1688 cells treated with 3 μM SAHA for different days(**D**).

**Supplement 2.** SAHA induces CCF in H1688 cells. (**A**) H1688 cells were treated with DMSO or 3 μM SAHA for six days. Immunofluorescent images stained with γΗ2ΑΧ and 53BP1 were performed at SAHA withdrawal two days later. Results were evaluated by the average values of five different fields with over 100 cells. Scale bars=10 µm. n=3 independent experiments. (**B**) The proportion of CCF was assessed as the average of five different fields over 100 cells in H1688 cells. The cells were treated in the same way as A, and then CCF was detected by immunofluorescence assay at SAHA withdrawal two days later. Error bars indicate Mean±SD, Student’s t-test. n=3 independent experiments. (**C**) The expression of STING protein in several cancer cell lines. The expression level of STING protein was evaluated using western blotting. (**D**) The expression levels of cGAS-STING pathway-related proteins in H1688 cells were determined after treatment of 3 μM SAHA and 10 μM cGAS inhibitor PF06928215 using western blotting. As the above method, H1688 cells were exposed to 3 μM SAHA for six days, and 10 μM PF06928215 treated for 24h after SAHA withdrawal two days later. For positive control of the cGAS-STING activation, H1688 cells were treated with 5 µM cGAMP for 4 h. (**E**) As the above method, H1688 cells were exposed to 3 μM SAHA for six days, and 1 μM C176 was treated for 48 h after SAHA withdrawal two days later, then determined by western blotting. **, *P* < 0.01; ***, *P* < 0.001 compared with the corresponding control.

**Supplement** **3.** Nuclear pore density increases following SAHA treatment in H1688 cells. (**A**) H1688 cells were treated with DMSO or 3 μM SAHA for six days, The relative mRNA level of Tpr was performed at SAHA withdrawal two days later by RT-PCR. Error bars represent Mean±SD, and the results are the average of three different experiments. mRNA levels were quantified relative to β**-**actin (control) mRNA. (**B**) Western blotting showed the expression levels of Tpr in H1688 cells treated with the same method as in experiment **A**. (**C**) H1688 cells were treated with the above method before immunofluorescence staining with Tpr, evaluating the change of nucleoporin Tpr area. Scale bars=5 µm, 10 µm. n=3 independent experiments. (**D**) H1688 cells transfected with a shTpr plasmid were treated with DMSO or 3 μM SAHA for six days and measured at SAHA withdrawal two days later by western blotting. (**E**) H1688 cells transfected with a shTpr plasmid were treated with DMSO or 3 μM SAHA for six days and the proportion of CCF was assessed as the average of five different fields over 100 cells by immunofluorescence staining with γΗ2ΑΧ and H3K27me3 at two days after withdrawal of SAHA. Error bars indicate Mean±SD. n=3 independent experiments. (**F**) The protein levels of cGAS-STING pathway and senescence-associated proteins in H1688 cells transfected with a shTpr plasmid were assessed by western blotting after processing according to the above method. *ns*, no significant; *, *P* < 0.05; **, *P* < 0.01; ***, *P* < 0.001 compared with the corresponding control.

**Supplement 4.** The combined treatment of SAHA and EZH2 inhibitor EPZ-6438 suppresses SAHA-induced SASP in SCLC cells. (**A**) EZH2 protein levels were evaluated by Western blotting in SCLC cell lines. (**B-C**) Western blotting determined the reasonable concentration of EPZ-6438 with 3 μM SAHA in H446 cells (**B**) and H1688 cells (**C**). H446 cells and H1688 cells were treated with DMSO or 3 μM SAHA and various concentrations of EZP-6438 and analyzed six days later. In H1688 cells, 3 μM SAHA was treated in a separate SAHA treatment area for six days, and was used for detection after two days. β-actin and H3 proteins are used as the loading control. (**D**) Western blotting was used to assess the expression levels of senescence-associated proteins in H1688 cells treated with DMSO or 3 μM SAHA and 0.5 μM EZP-6438 for six days. (**E**) The mRNA levels of IL6 and IL8 in H1688 cells after treatment of DMSO or 3μ M SAHA and 0.5 μM EZP-6438 for six days were detected using RT-PCR. mRNA levels were quantified relative to β**-**actin (control) mRNA and represented as Mean±SD of three independent experiments. In H1688 cells, 3 μM SAHA was treated in a separate SAHA treatment area for six days, and was used for detection after two days. (**F**) shEZH2 plasmid was transfected into H1688 cells. RT-PCR was used to examine the mRNA levels of IL6 and IL8 after treatment of DMSO or 3 μM SAHA for six days in H1688 cells transfected with a shEZH2 plasmid. H1688 cells transfected with a pLKO plasmid were treated with 3 μM SAHA for six days and used to assess the mRNA levels of IL6 and IL8 after SAHA withdrawal for two days. n=3 independent experiments. (**G**) The expression levels of senescence-associated proteins in H1688 cells transfected with a shEZH2 plasmid were assessed after treatment of DMSO or 3 μM SAHA for six days by western blotting. H1688 cells transfected with a pLKO plasmid were treated with the above method and used for western blotting. (**H**) H1688 cells were treated with DMSO or 3 μM SAHA and 0.5 μM EZP-6438 for six days and subsequently stained for SA-β-gal after SAHA withdrawal two days later in the SAHA alone treatment group. The result is the percentage of the positive cells relative to cells detected, repeated three times independently. (**I**) Immunofluorescent images were used to assess the senescent state of H1688 cells treated with DMSO or 3 μM SAHA and 0.5 μM EZP-6438 for six days. H1688 cells were treated with 3 μM SAHA in a separate SAHA treatment area for six days, and were used for detection after two days. Scale bars=10 µm. n=3 independent experiments. (**J**) Results are the percentage of positive cells over 100 cells in H1688 cells. n=3 independent experiments. *ns*, no significant; *, *P* < 0.05;**, *P* < 0.01; ***, *P* < 0.001 compared with the corresponding control.

**Supplement 5.** The inhibition of SASP enhances the antiproliferative effect of SAHA in SCLC cells. (**A**) The colony formation rate was assessed as the percentage of colonies formed in the presence of a normal medium after treatment of 3 μM SAHA, the combination of 3 μM SAHA and 0.5 μM EZP-6438 for six days in H1688 cells. The colony formation assay was performed ten days after drug withdrawal. n=3 independent experiments. (**B**) H1688 cells transfected with a shEZH2 plasmid were treated with 3 μM SAHA for six days and the colony formation rate was detected after incubating in a normal medium. The colony formation assay was conducted on the same day. n=3 independent experiments. (**C-D**) H446 cells (**C**) and H1688 cells (**D**) were incubated in CM and were evaluated for proliferation rates using the MTT experiment every other day for eight days. n=3 independent experiments. (**E**) H1688 cells were treated with the combination of 3 μM SAHA and 0.5 μM EZP-6438 for six days and subsequently incubated for eight days in the presence of CM, the cell proliferation was examined using an MTT assay every other day. n=3 independent experiments. (**F**) H1688 cells were treated with the combination of 3 μM SAHA and 0.5 μM EZP-6438 for six days and subsequently incubated in the presence of CM, the colony formation rate was analyzed fifteen days later. The values of all experiments represent the Mean±SD of three independent experiments. *ns*, no significant; *, *P* < 0.05;**, *P* < 0.01; ***, *P* < 0.001 compared with the corresponding control.

**Supplement 6.** Combination therapy of SAHA with the EZH2 inhibitor EPZ-6438 improves survival in lung cancer patients. (**A-B**) The survival of human lung cancer patients negatively correlates with expression levels of SAHA targeting genes (**A**) and EZP-6438 targeting gene (**B**). Kaplan-Meier curves were obtained using online software (https://kmplot.com/analysis/ index.php).
