# Supplementary figures and images for: Inhibition of EZH2 alleviates SAHA-induced senescence-associated secretion phenotype in small cell lung cancer cells
Source: Cell Death Discov. 2023 Aug 5;9:289. doi: 10.1038/s41420-023-01591-y (PMC10404275; doi:10.1038/s41420-023-01591-y)

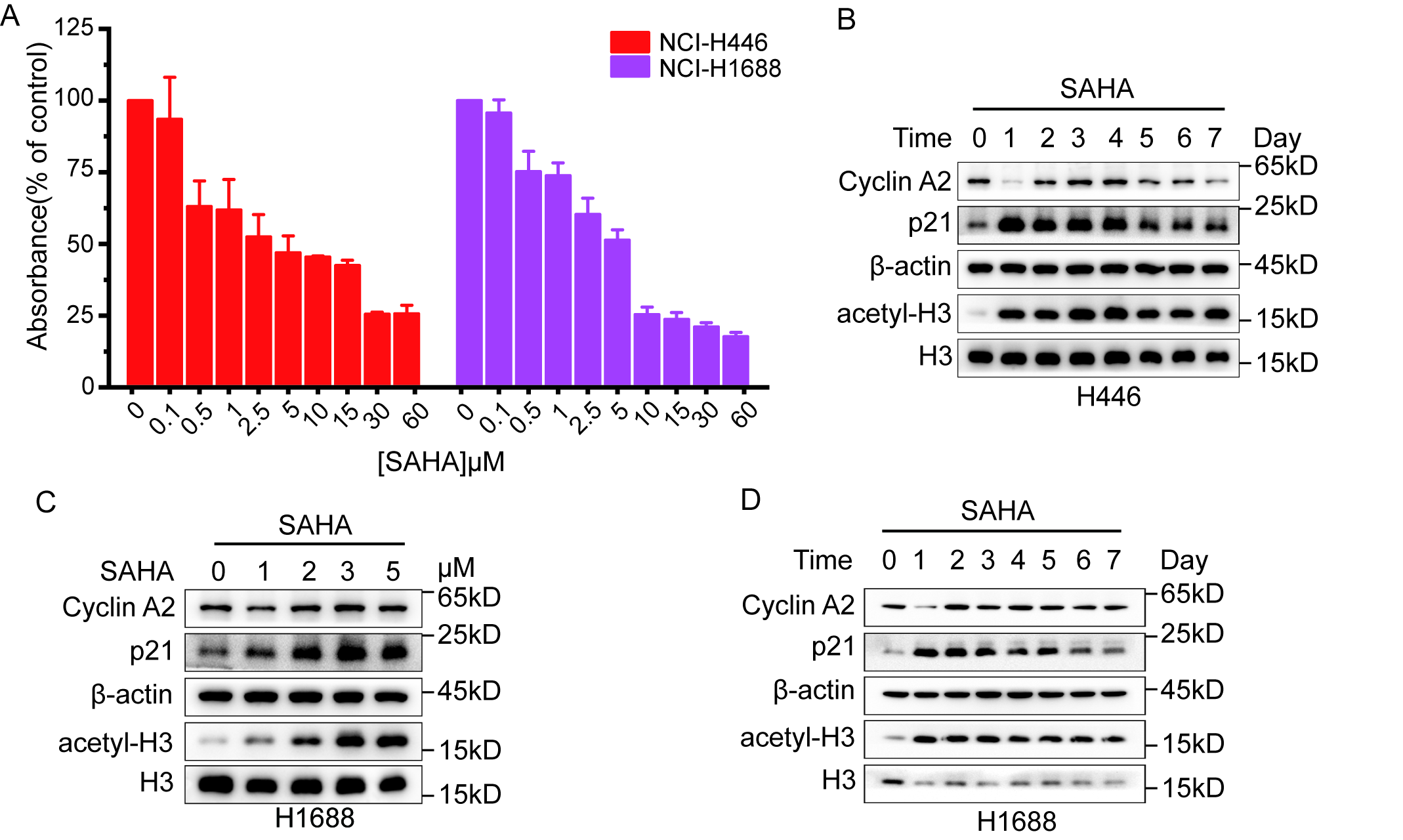

Supplement: Supplementary file 2 — Supplementary figure 1 [file 41420_2023_1591_MOESM2_ESM.tif]

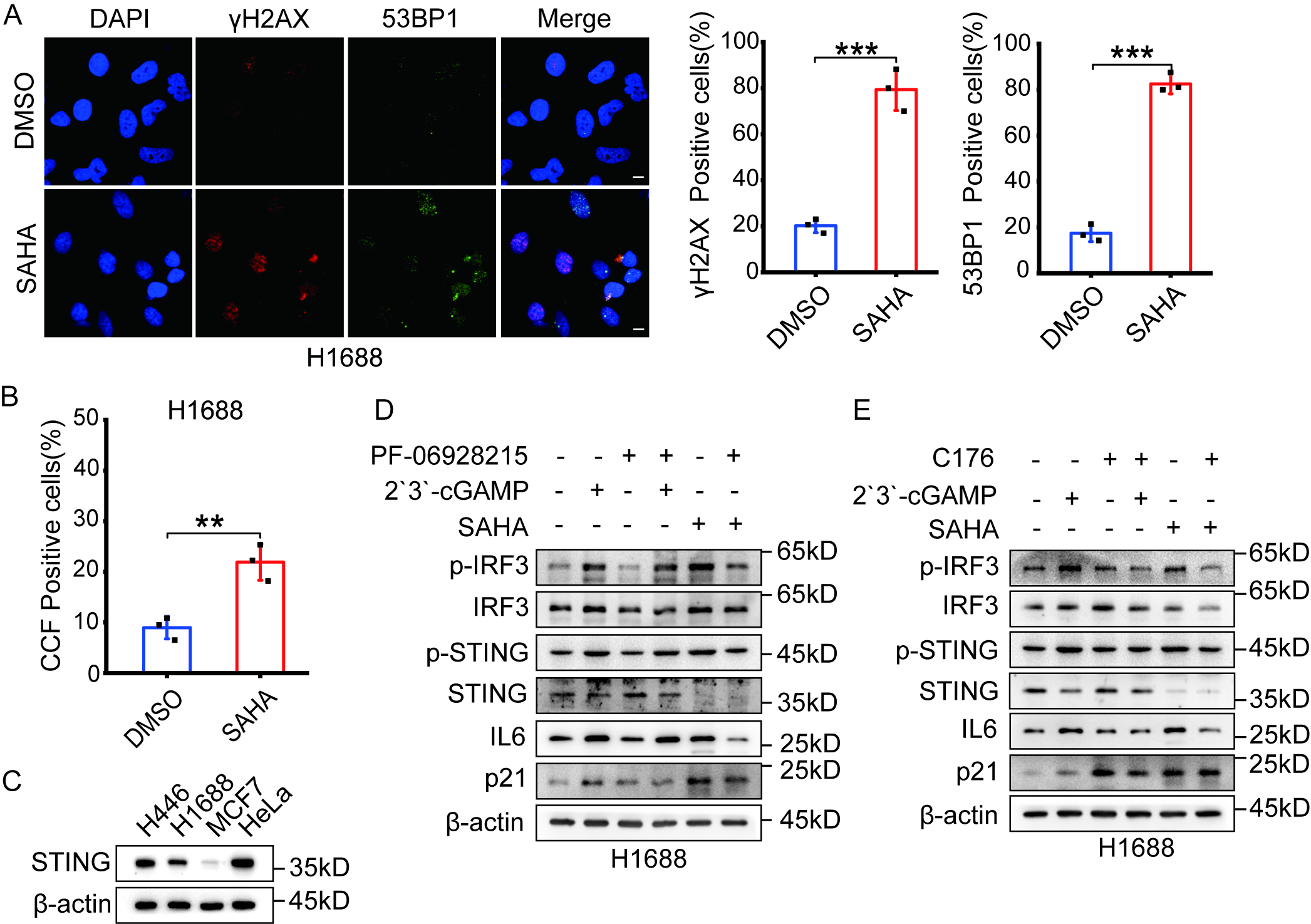

Supplement: Supplementary file 3 — Supplementary figure 2 [file 41420_2023_1591_MOESM3_ESM.tif]

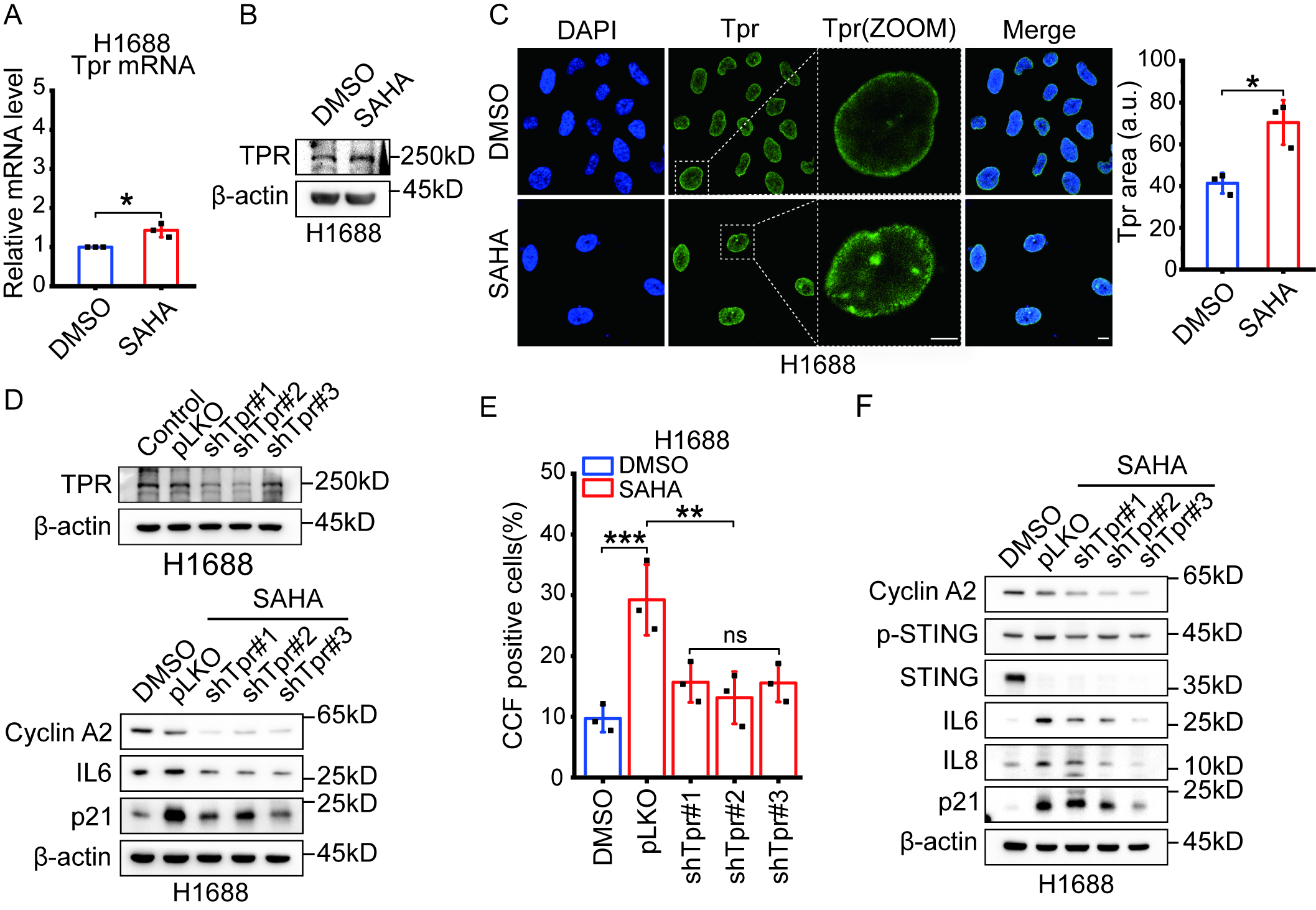

Supplement: Supplementary file 4 — Supplementary figure 3 [file 41420_2023_1591_MOESM4_ESM.tif]

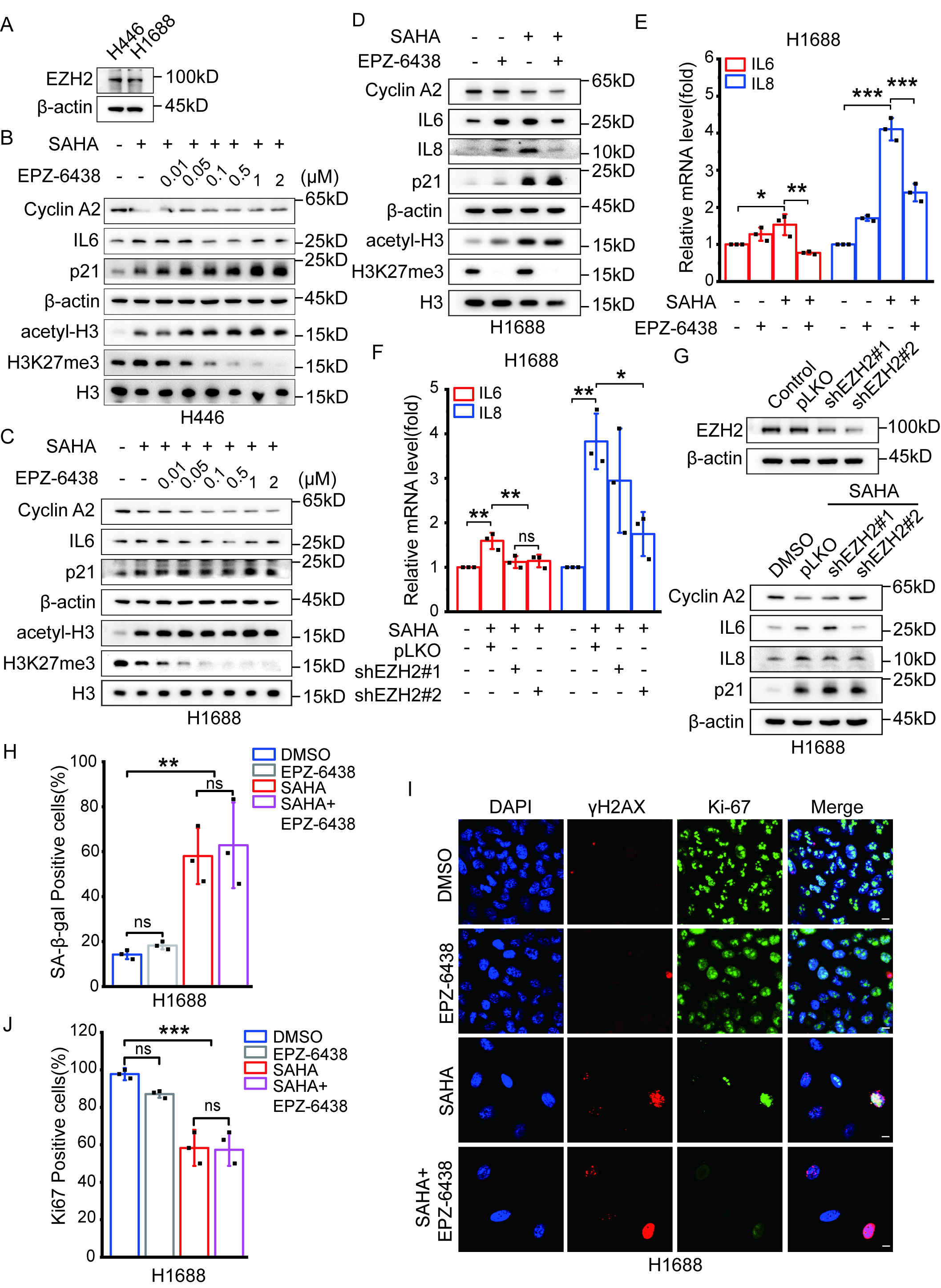

Supplement: Supplementary file 5 — Supplementary figure 4 [file 41420_2023_1591_MOESM5_ESM.tif]

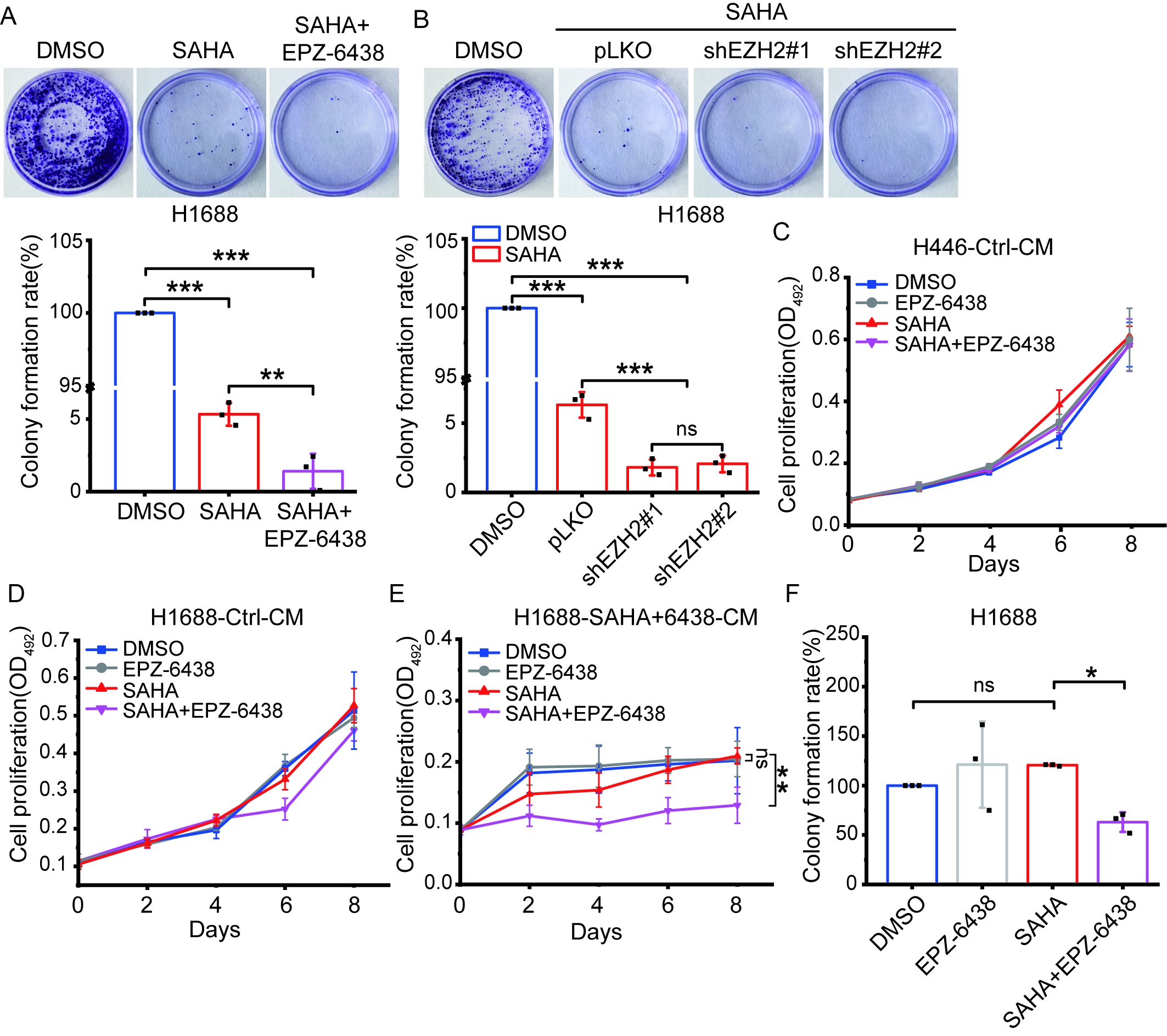

Supplement: Supplementary file 6 — Supplementary figure 5 [file 41420_2023_1591_MOESM6_ESM.tif]

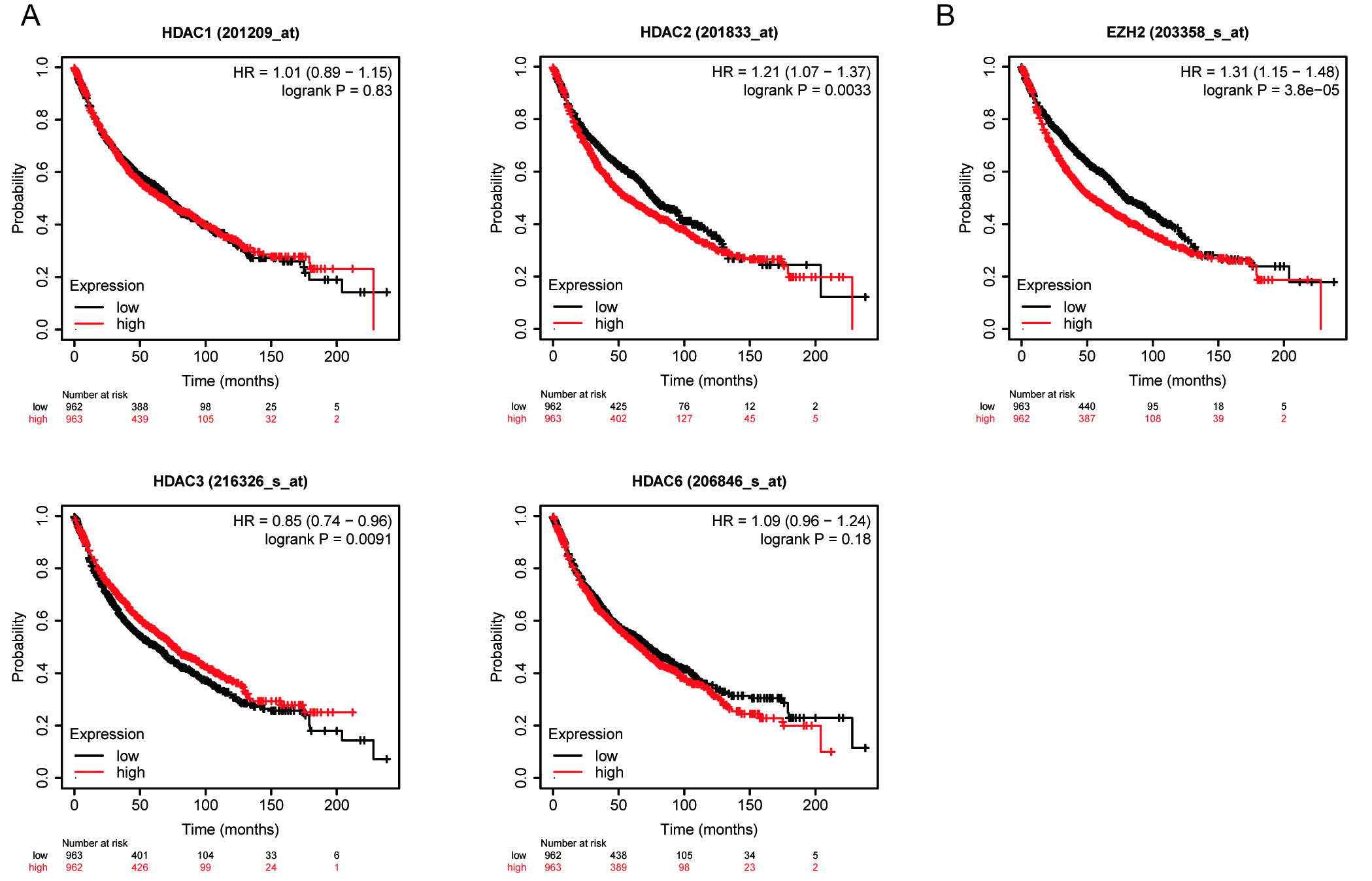

Supplement: Supplementary file 7 — Supplementary figure 6 [file 41420_2023_1591_MOESM7_ESM.tif]
