# Supplementary material for: Inhibition of EZH2 alleviates SAHA-induced senescence-associated secretion phenotype in small cell lung cancer cells
Source: Cell Death Discov. 2023 Aug 5;9:289. doi: 10.1038/s41420-023-01591-y (PMC10404275; doi:10.1038/s41420-023-01591-y)
Supplement: Supplementary file 11 — Original Data File [file 41420_2023_1591_MOESM11_ESM.pdf]

Figure 1A

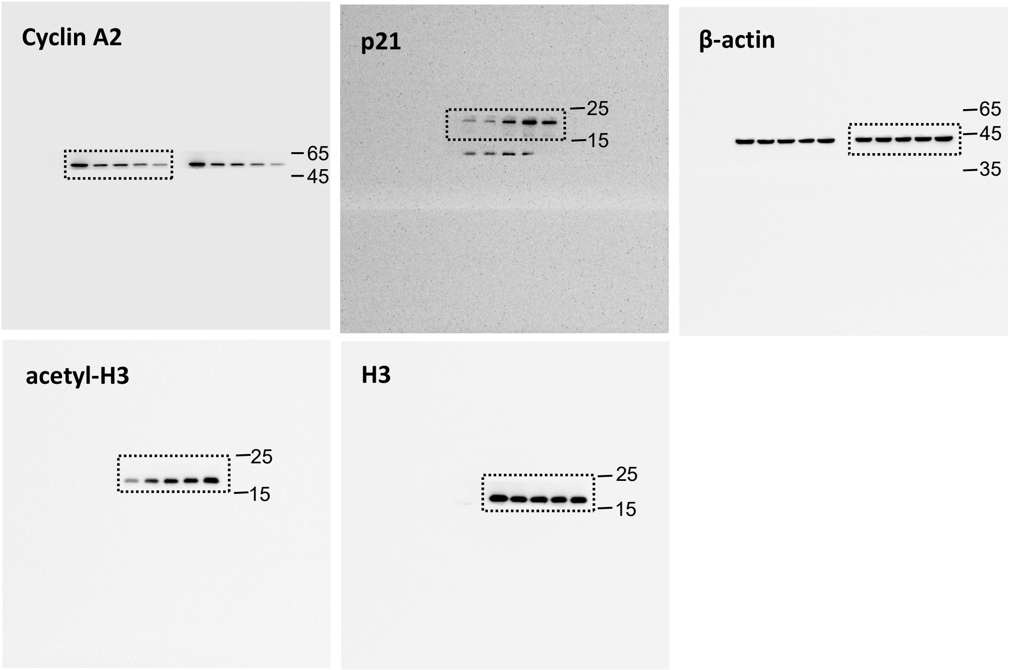

Figure 1C

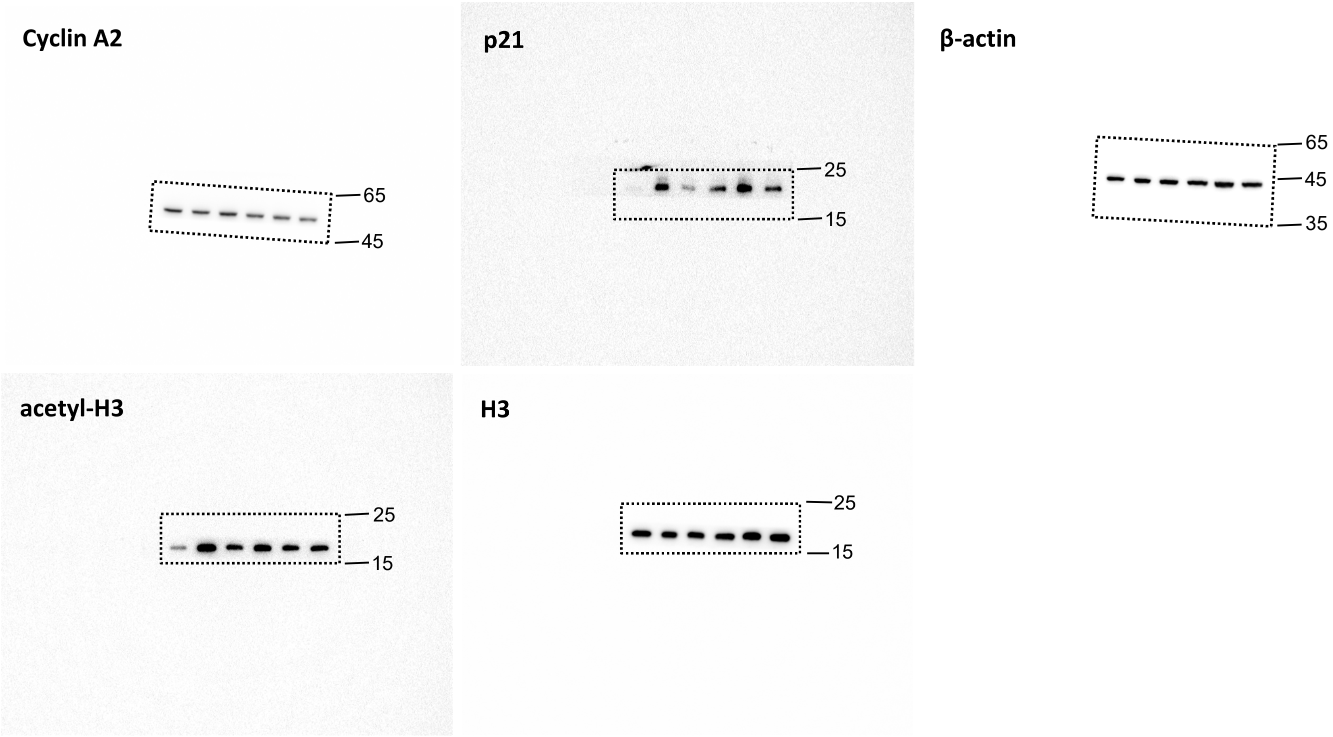

Figure 1K

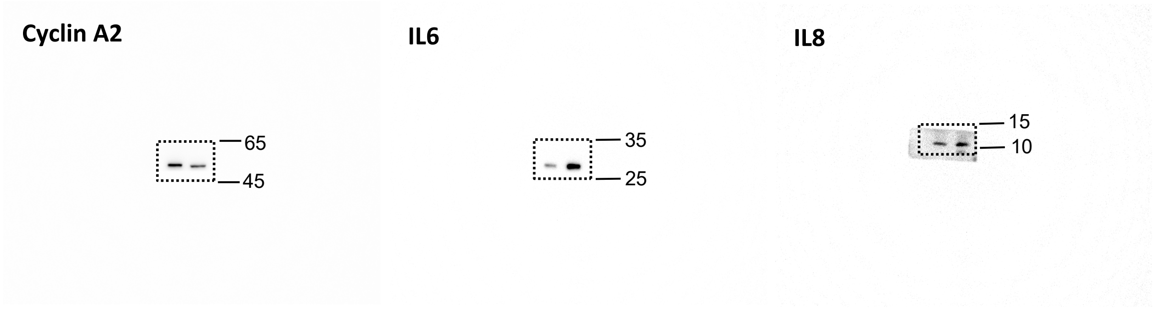

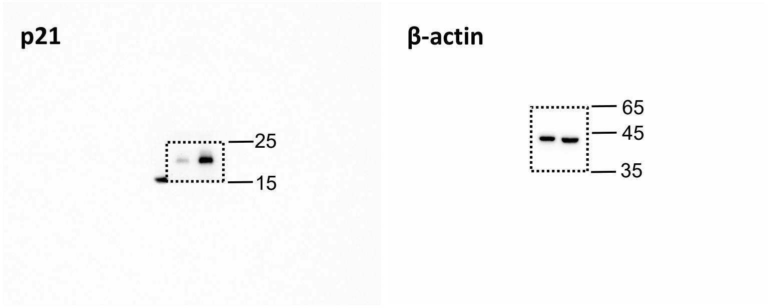

Figure 1L

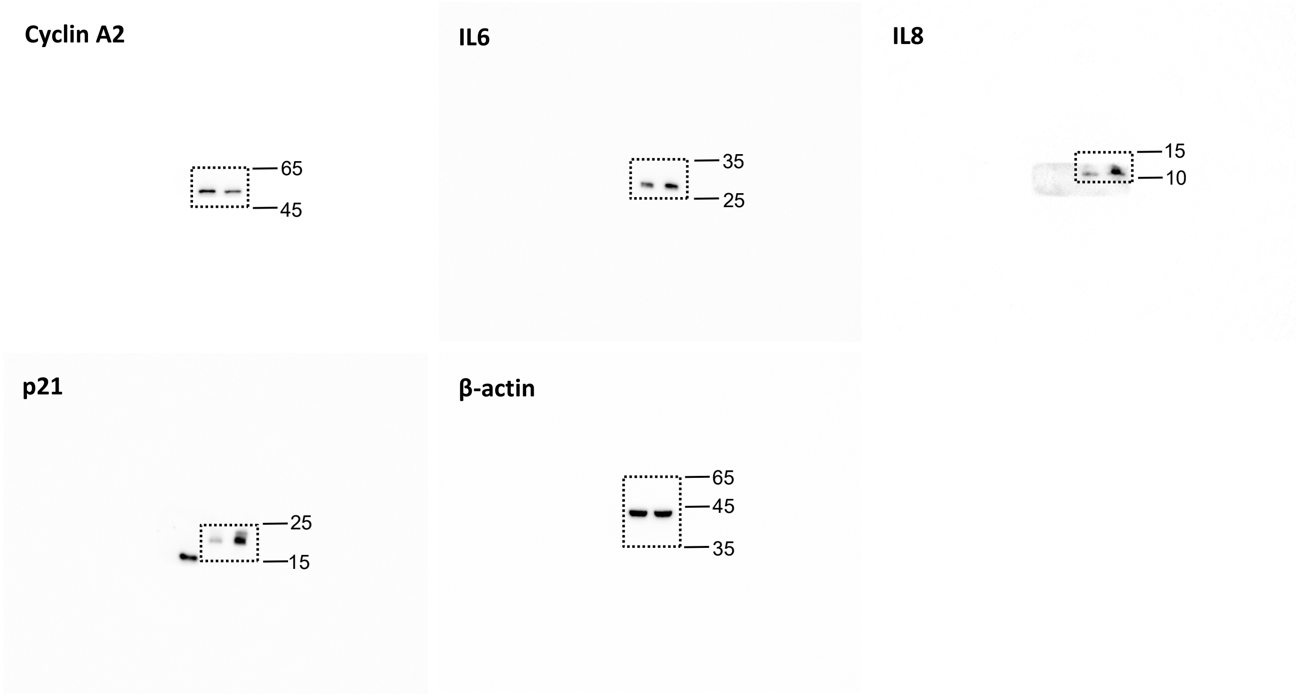

Figure 2D

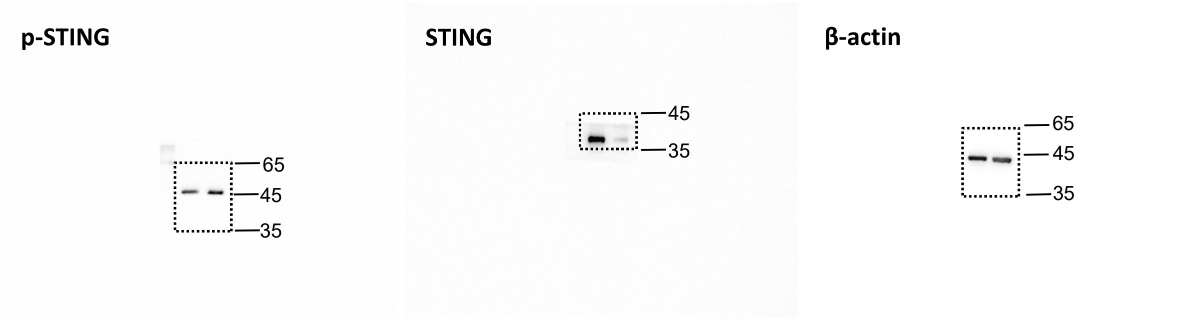

Figure 2E

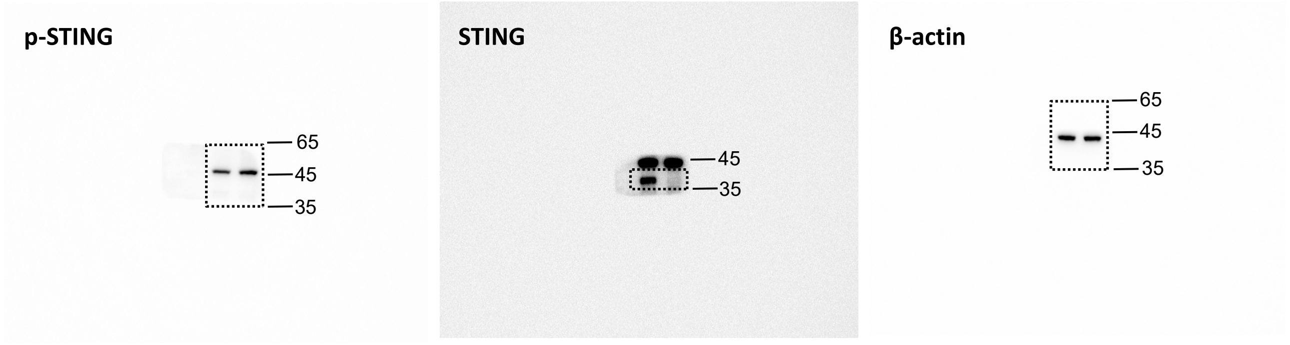

Figure 2F

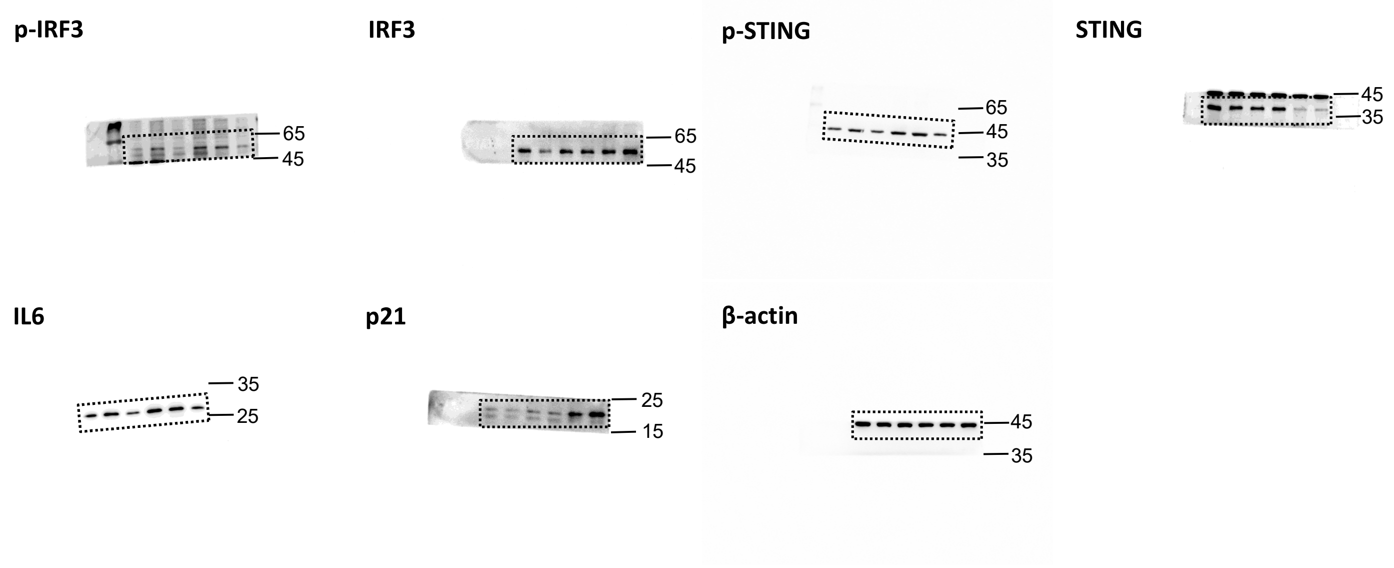

Figure 2G

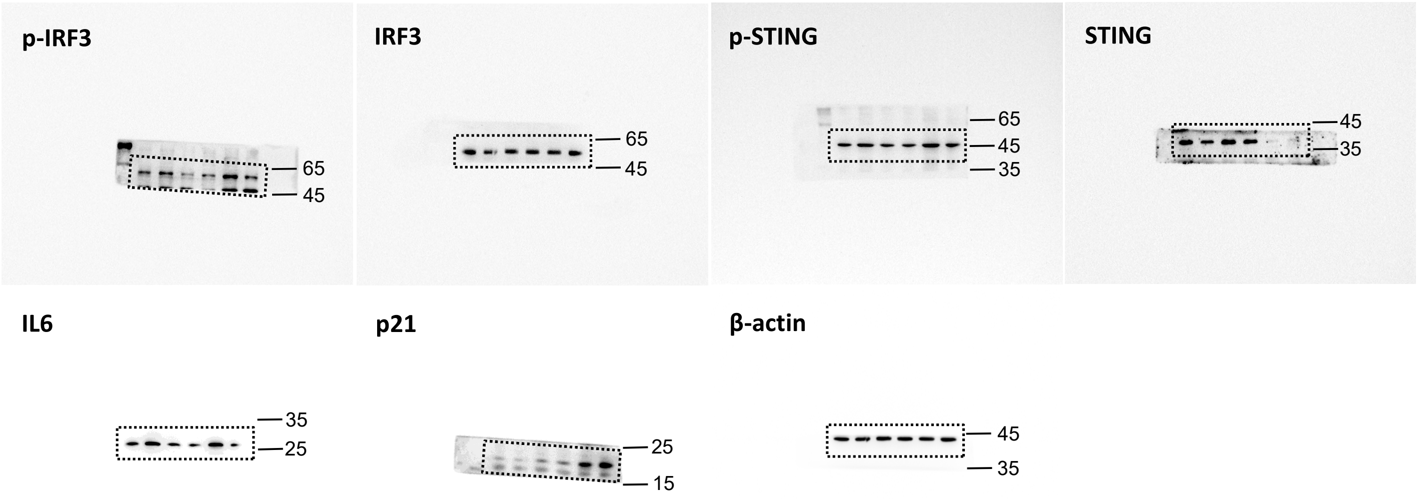

Figure 3B

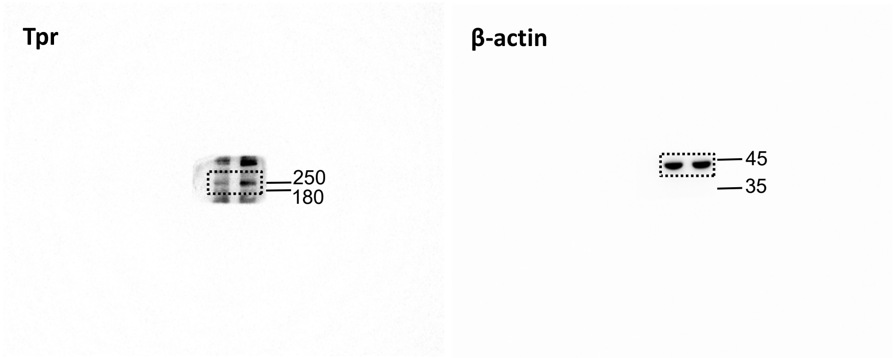

Figure 3D

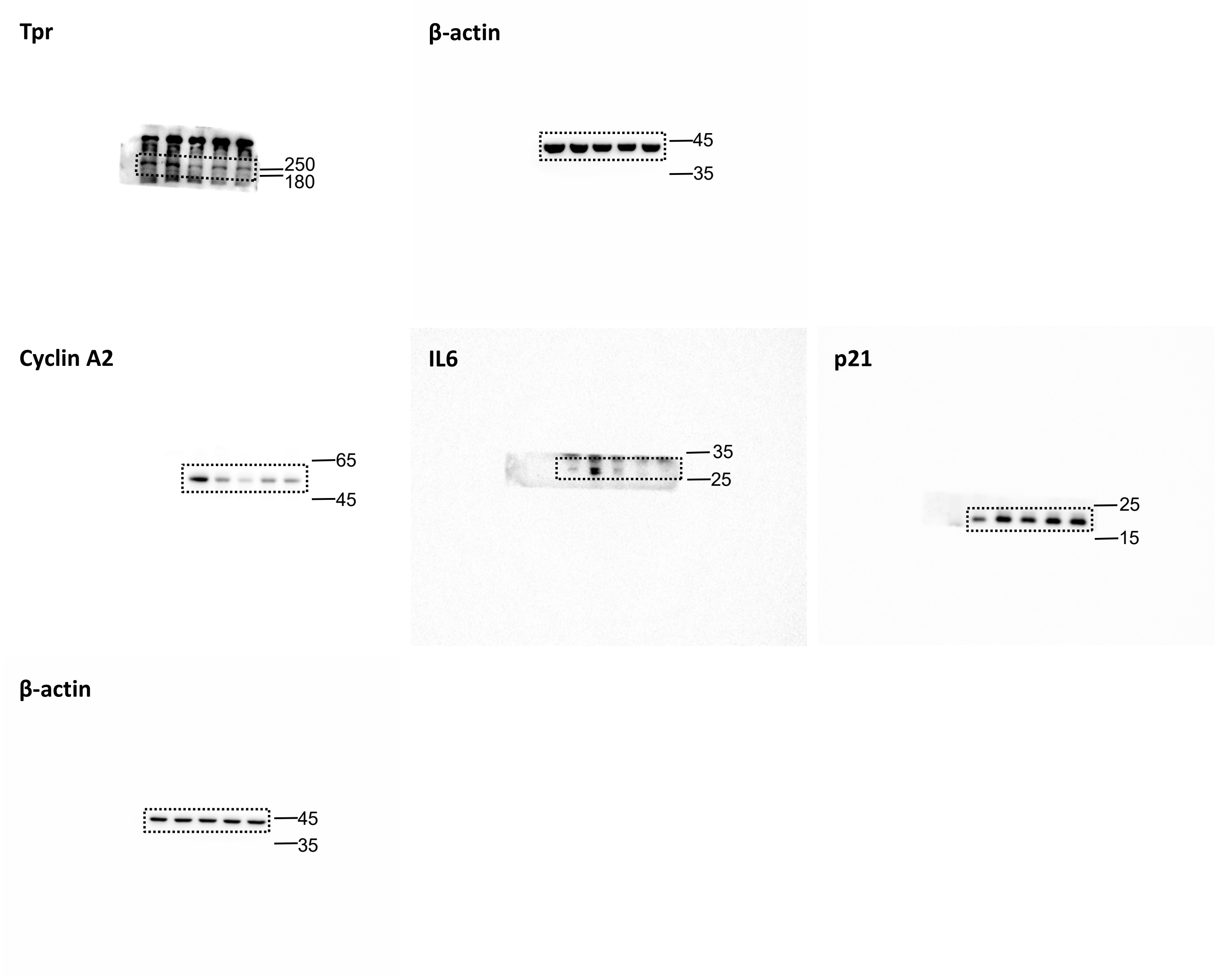

Figure 3G

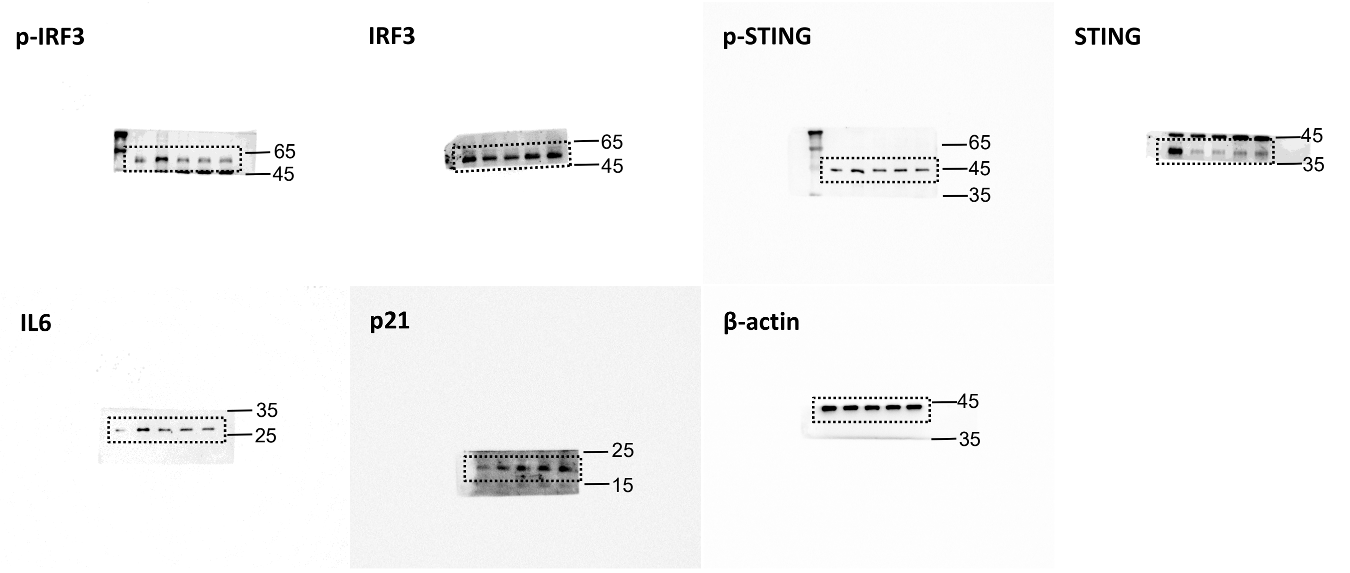

Figure 4A

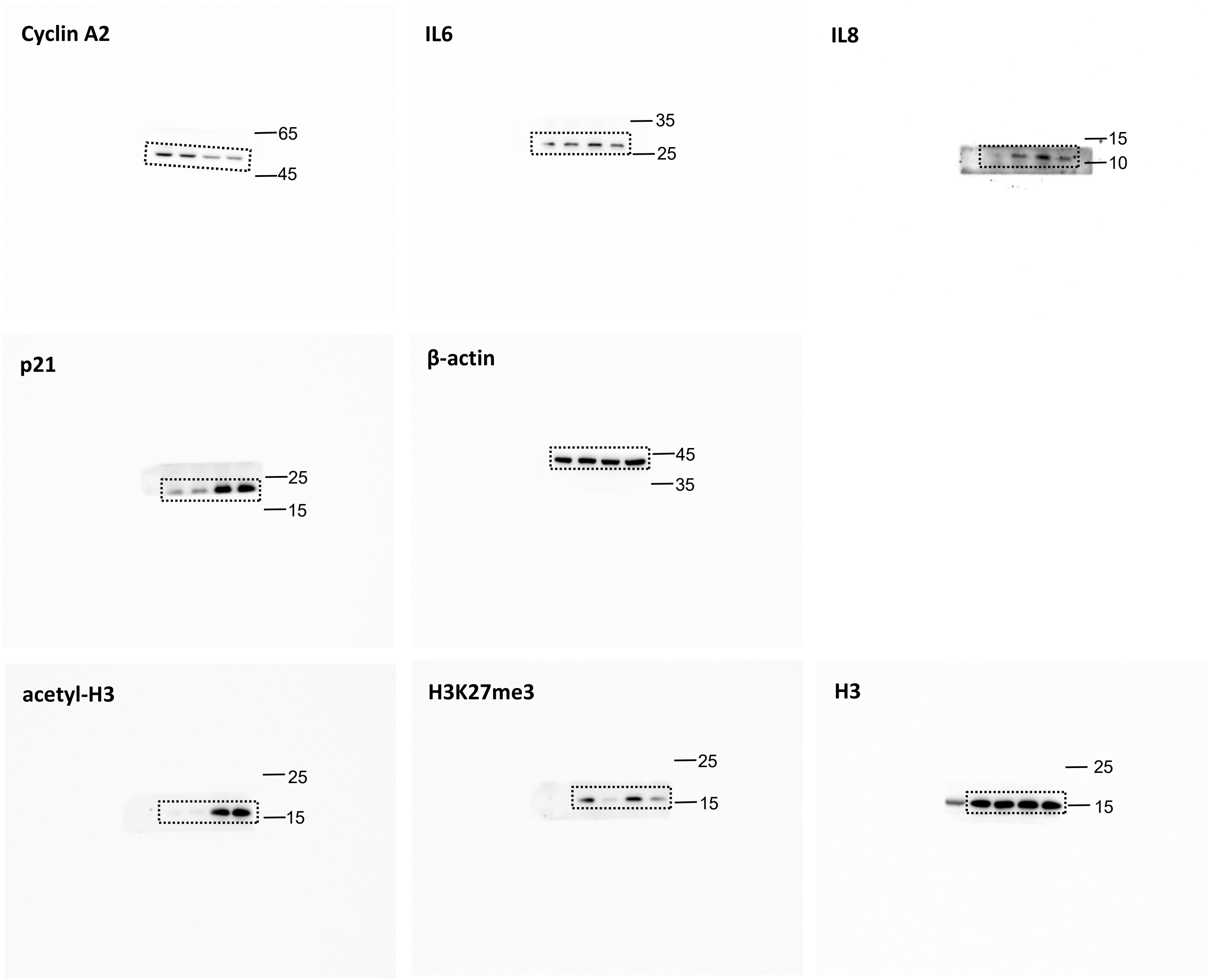

Figure 4D

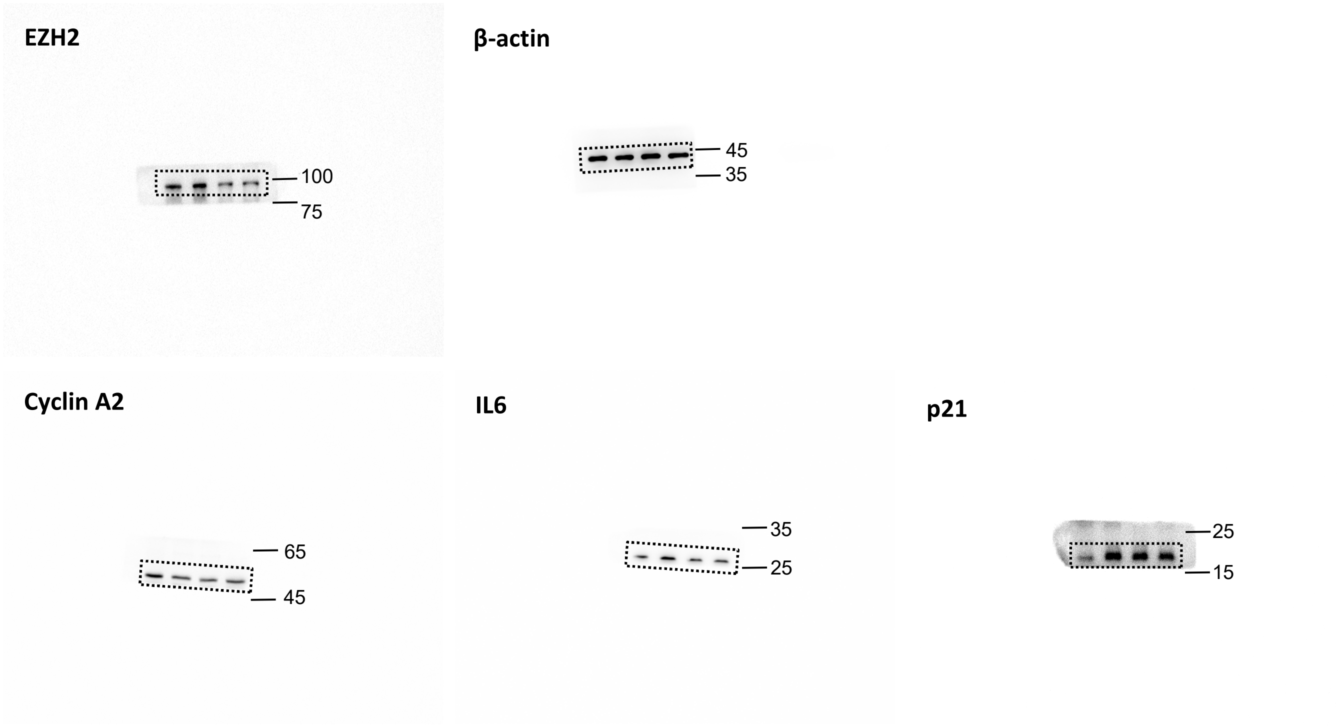

β-actin

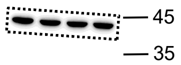

Figure 5C

p-IRF3

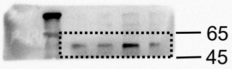

IRF3

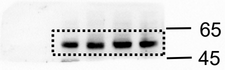

p-STING

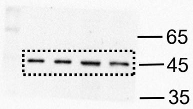

STING

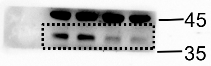

IL6

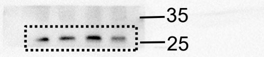

p21

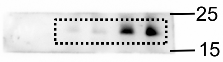

β-actin

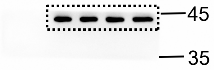

Figure 5D

p-IRF3

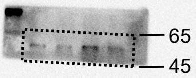

IRF3

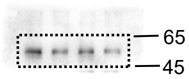

p-STING

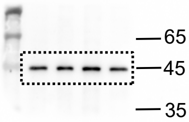

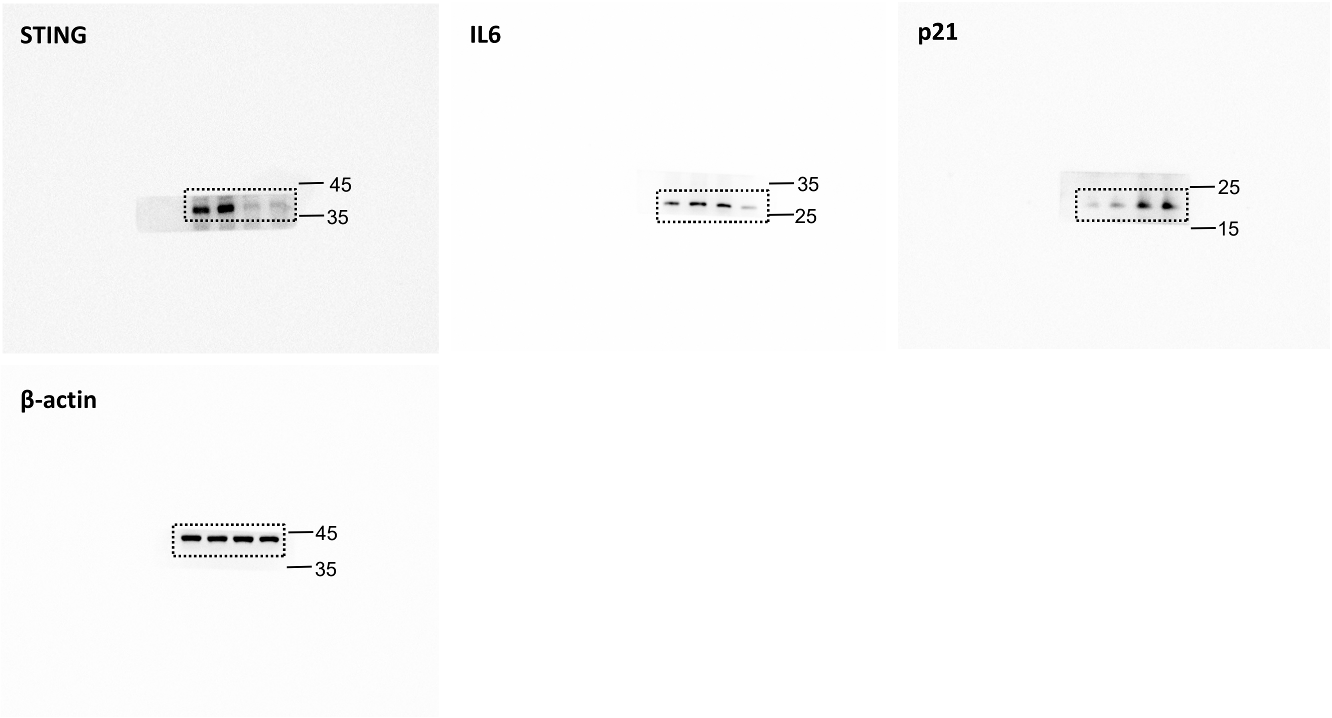
STING
45
35
IL6
35
25
p21
25
15
β-actin
45
35

Figure 5G

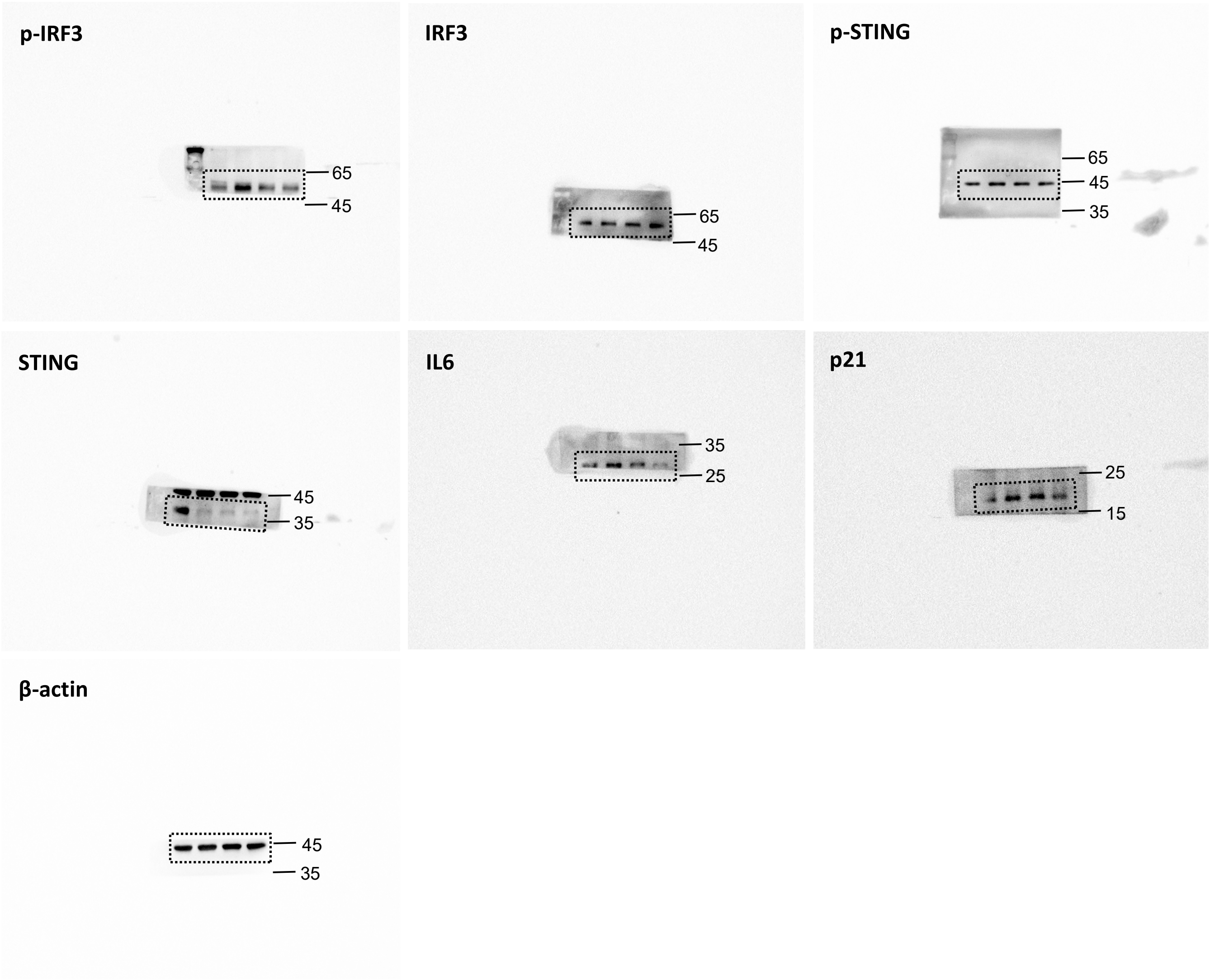
p-IRF3
65
45
IRF3
65
45
p-STING
65
45
35
STING
45
35
IL6
35
25
p21
25
15
β-actin
45
35

Figure 5H

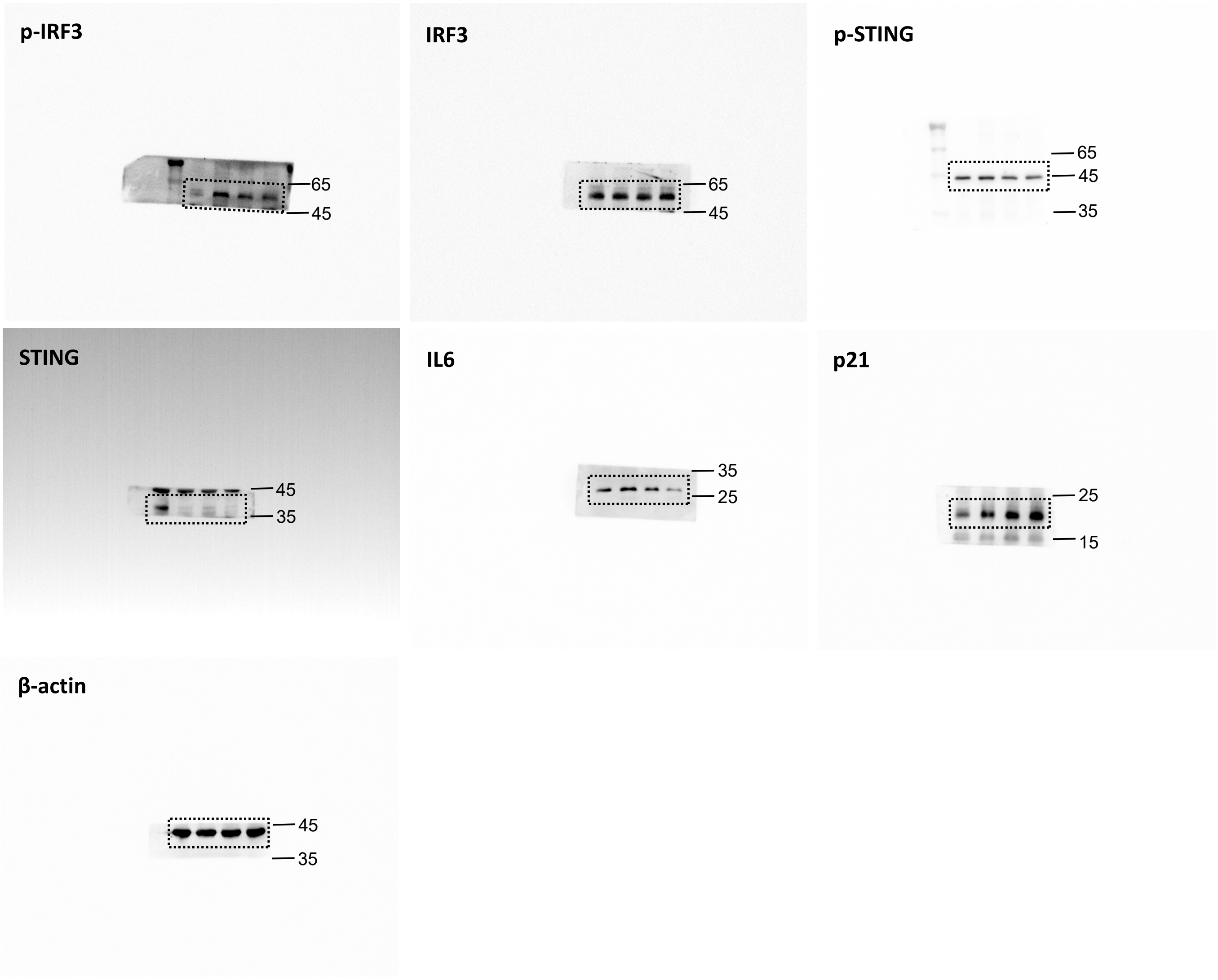

Figure S-1B

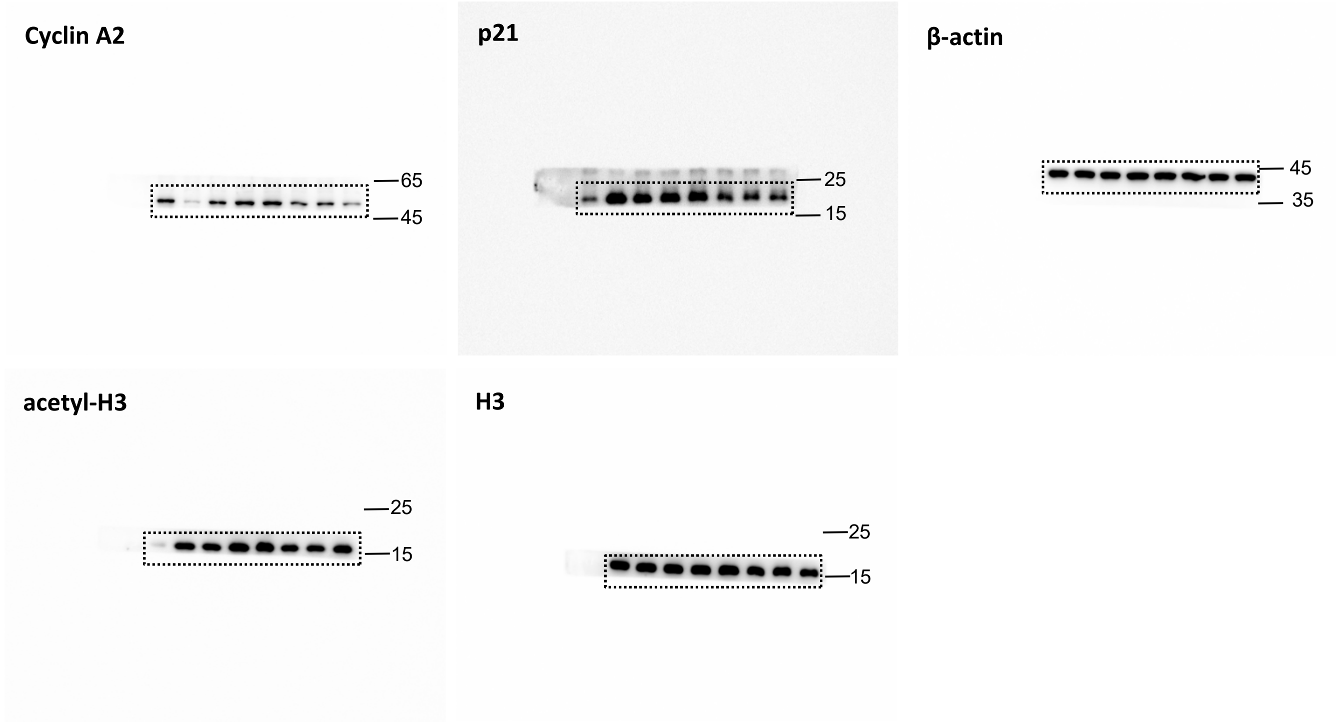

Figure S-1C

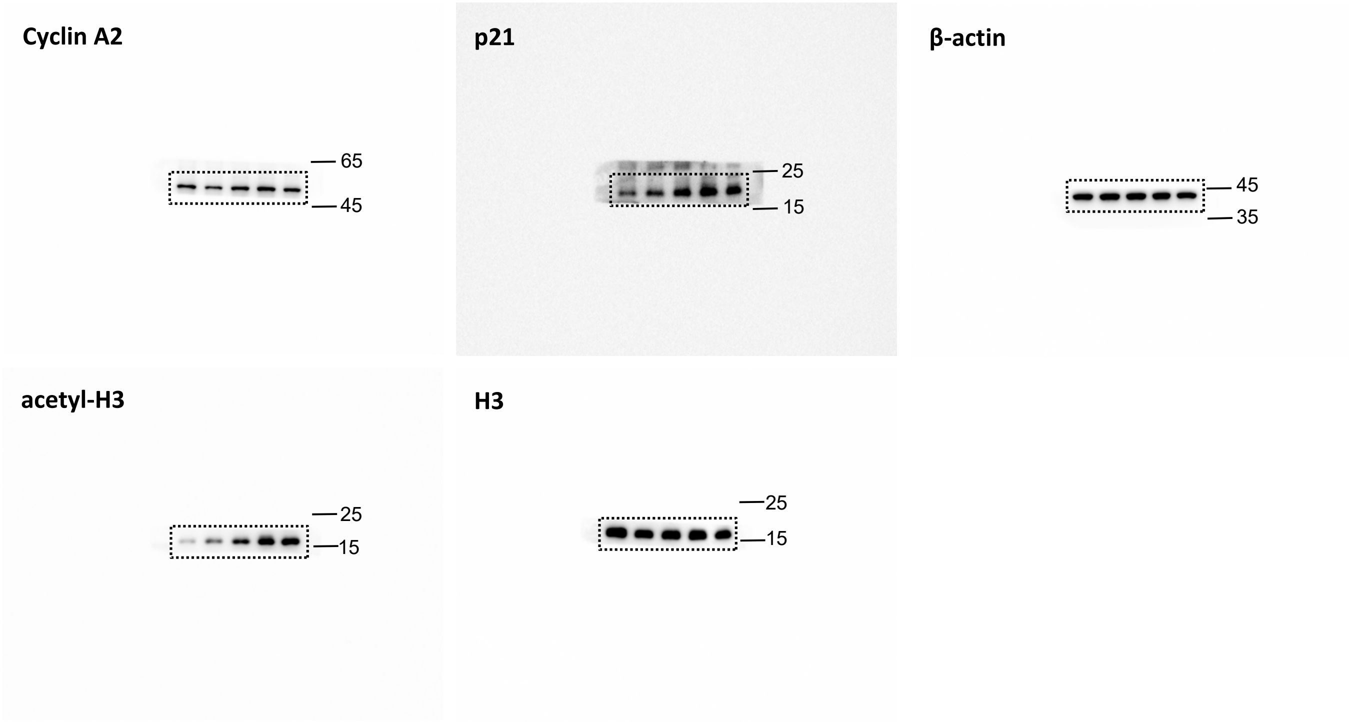

Figure S-1D

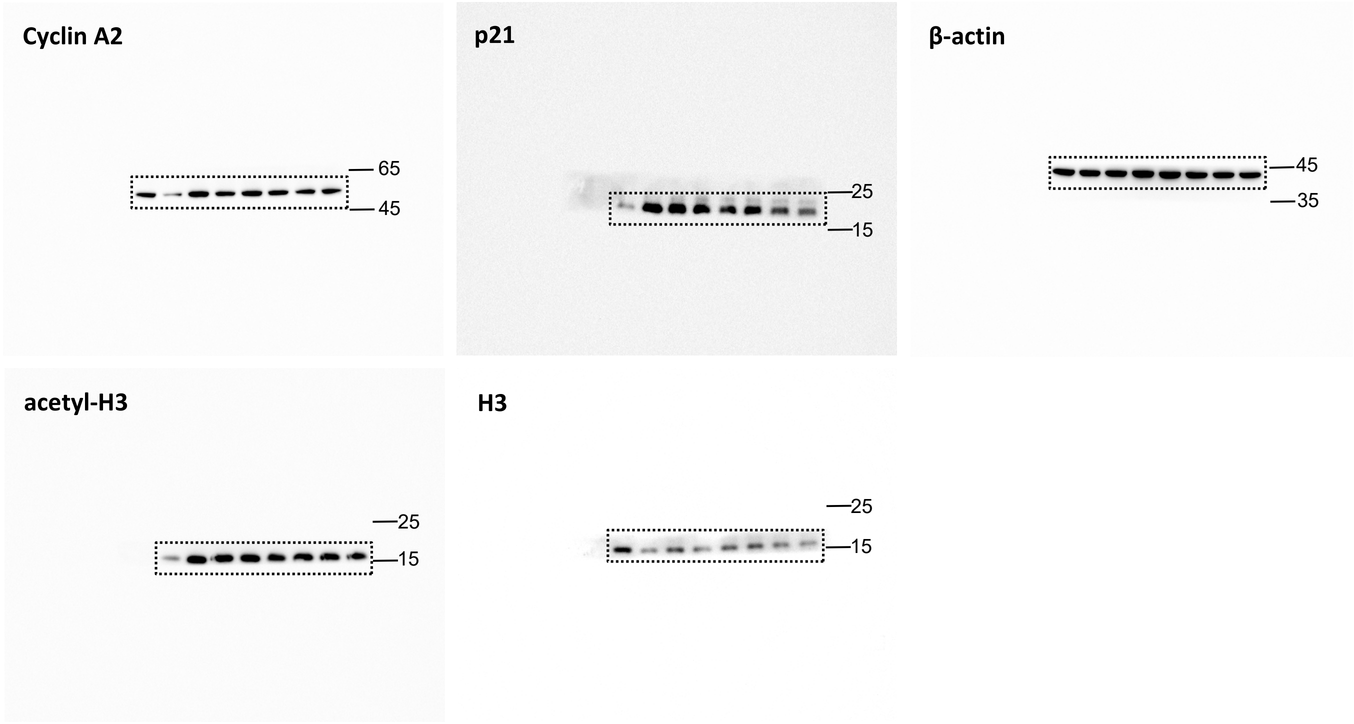

Figure S-2C

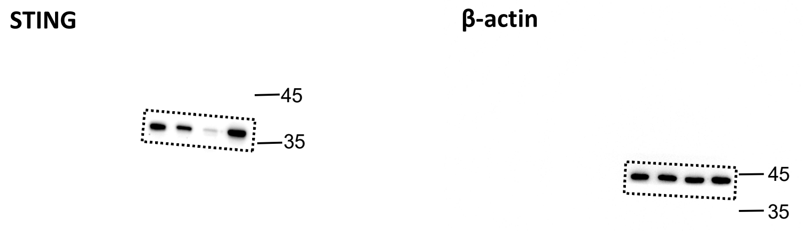

Figure S-2D

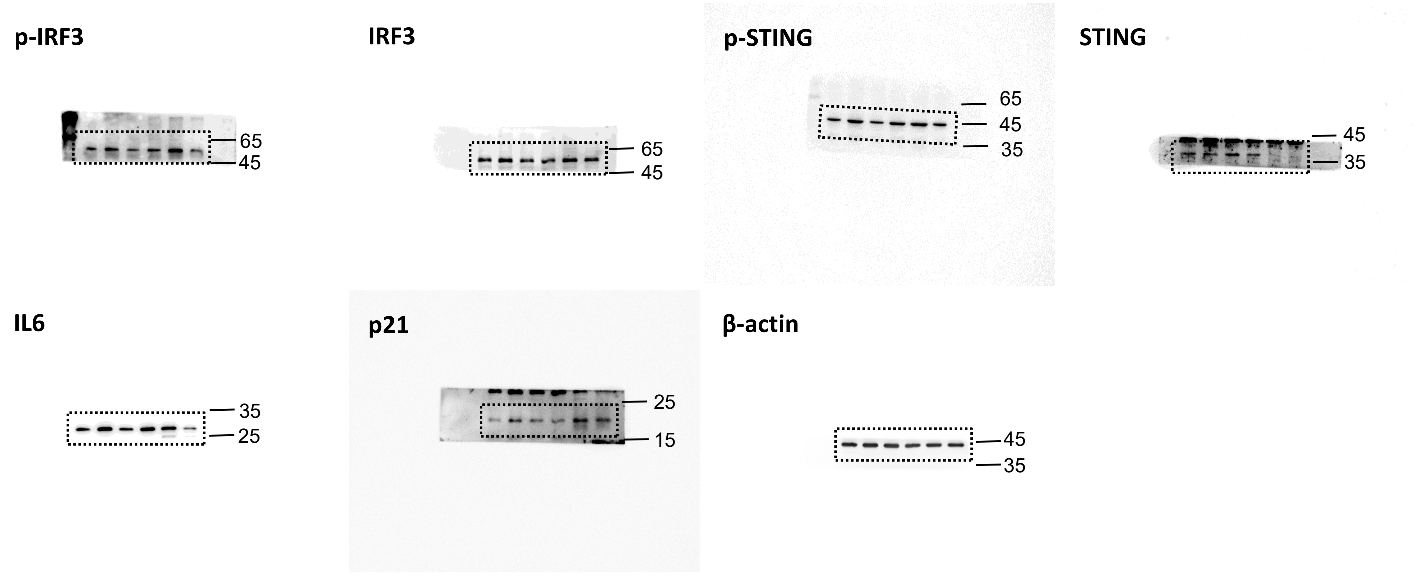

Figure S-2E

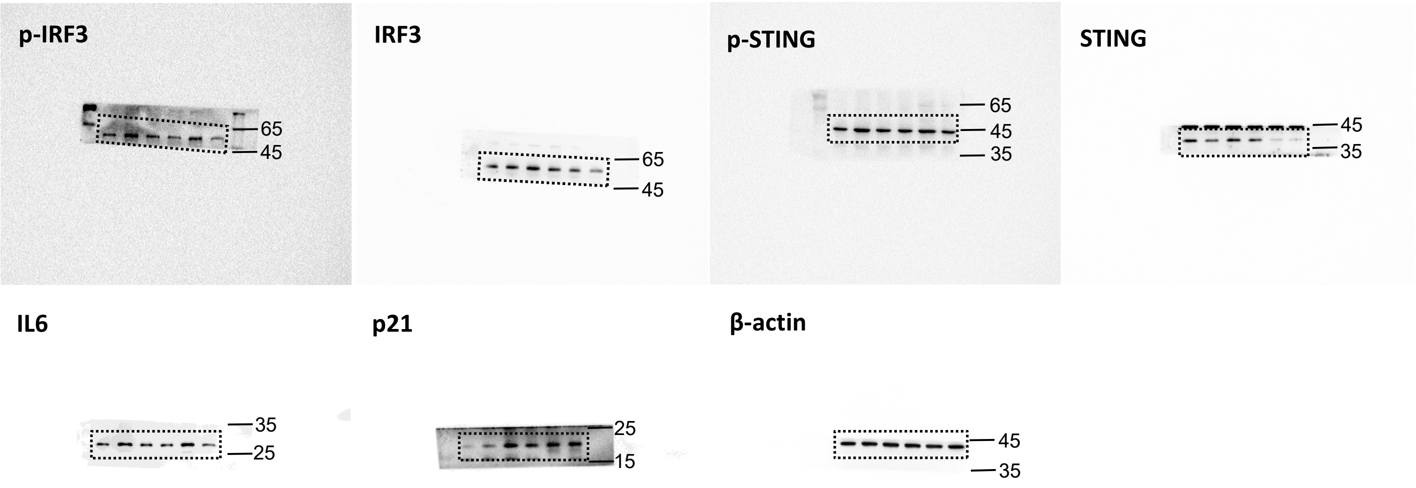

Figure S-3B

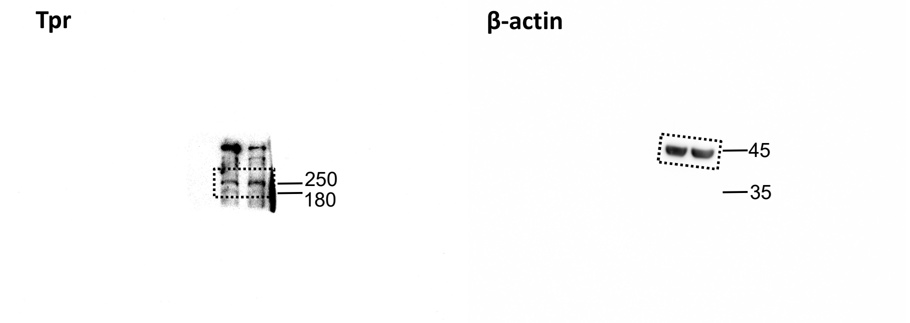

Figure S-3D

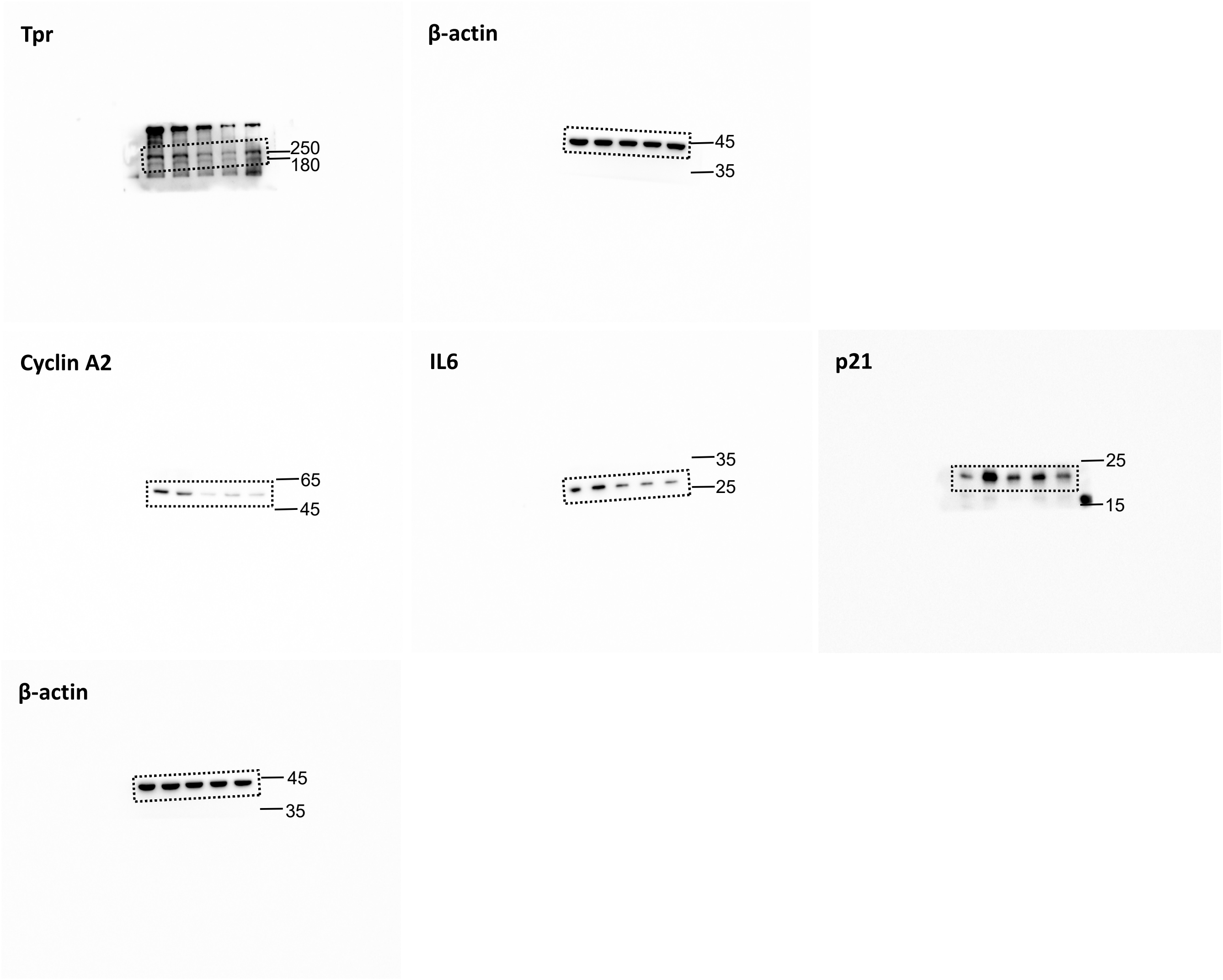

Figure S-3F

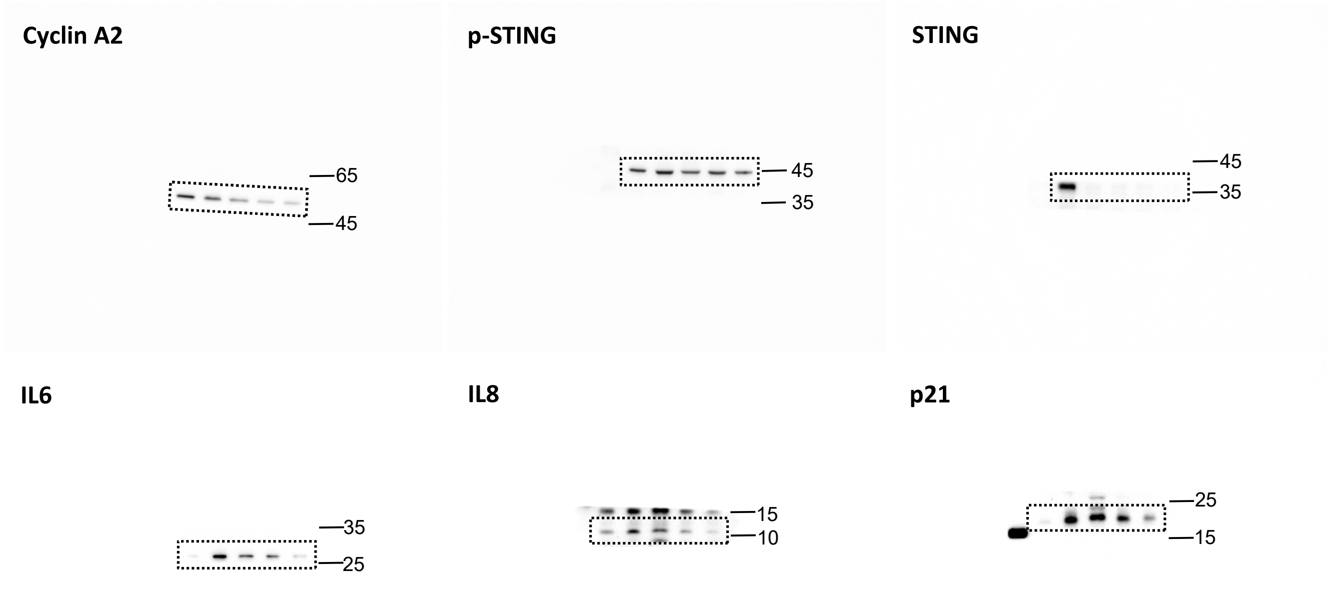

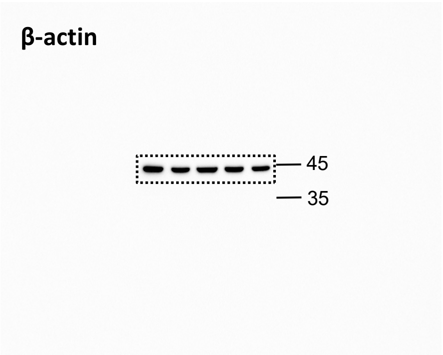

Figure S-4A

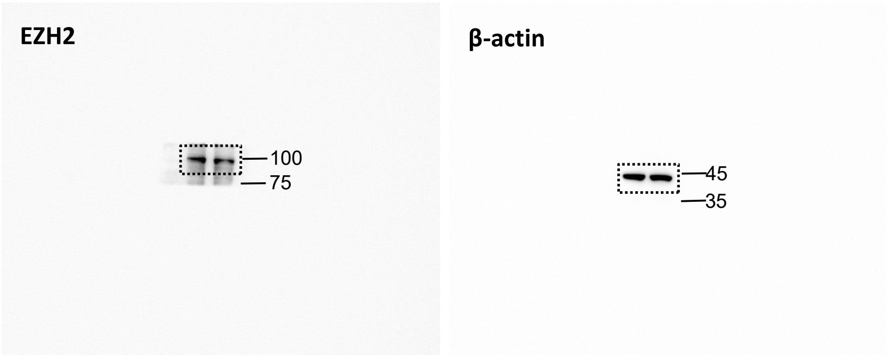

Figure S-4B

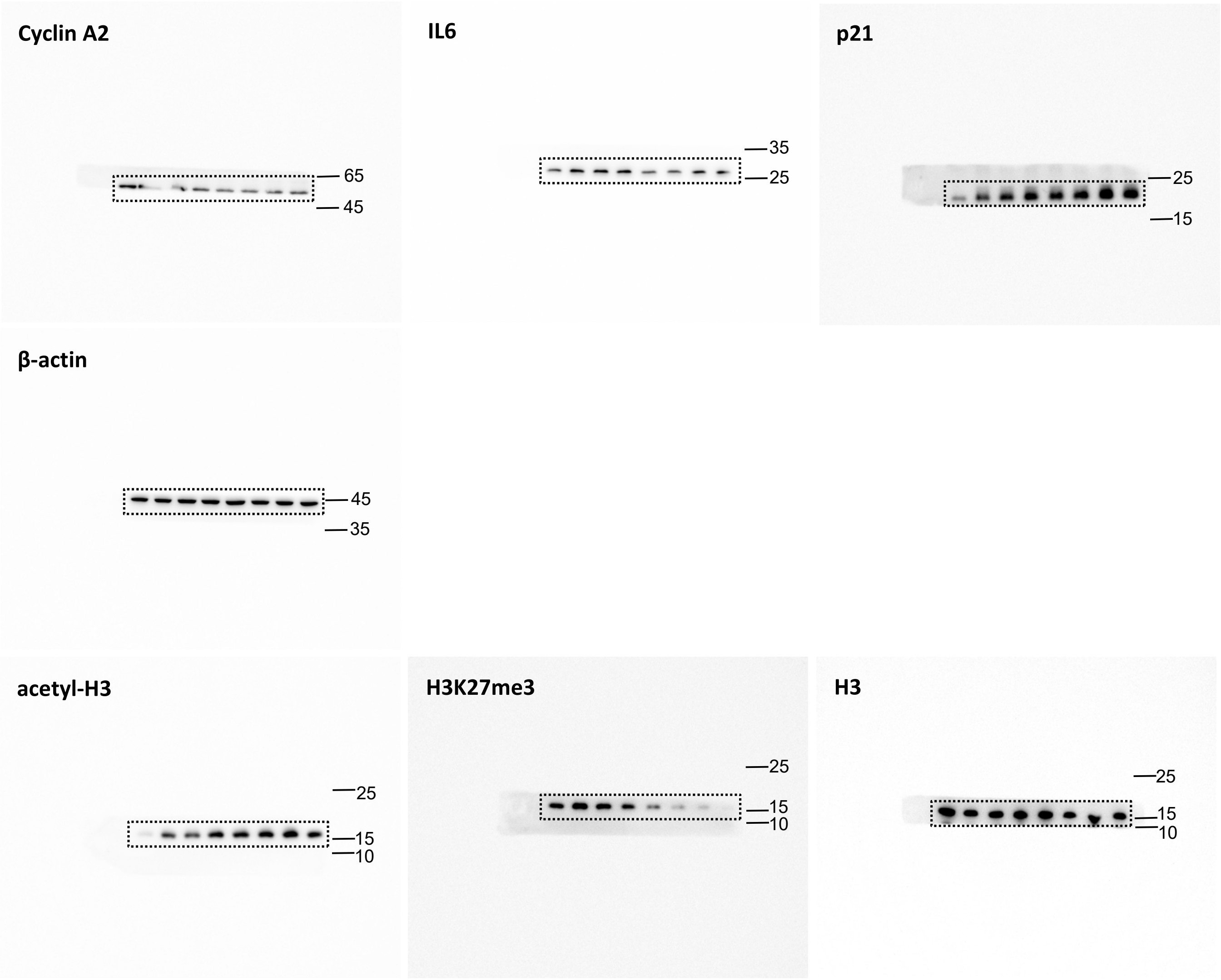

Figure S-4C

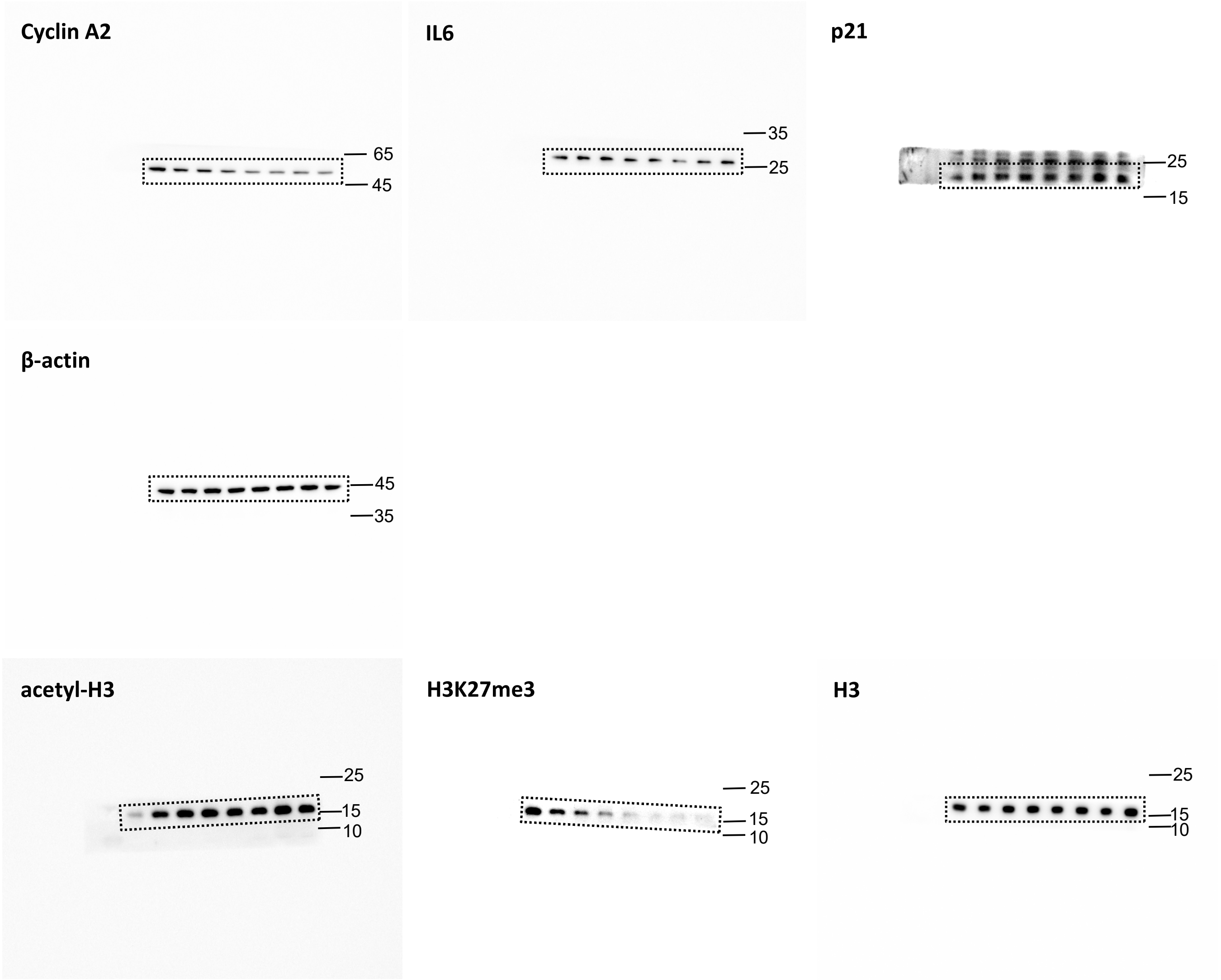

Figure S-4D

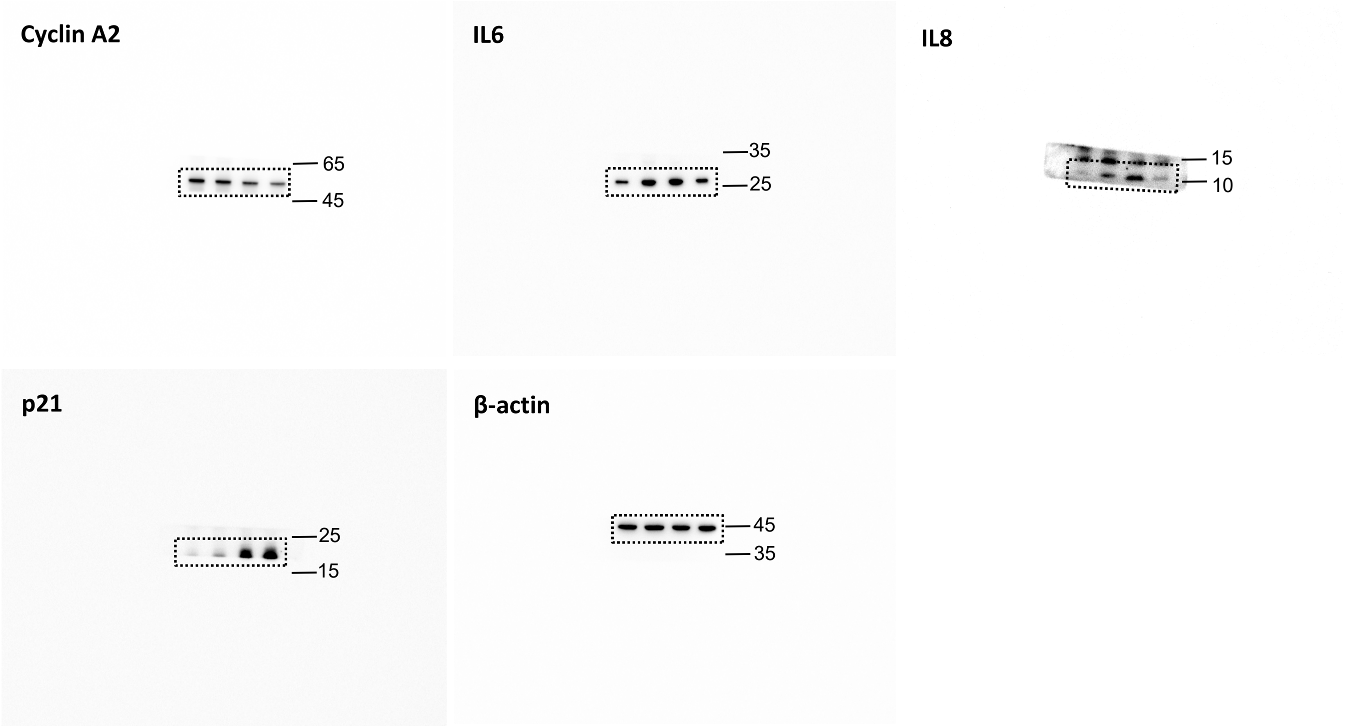

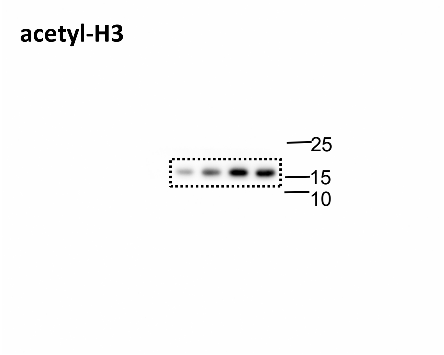
acetyl-H3
25
15
10

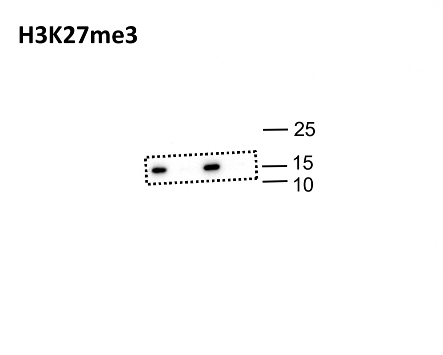
H3K27me3
25
15
10

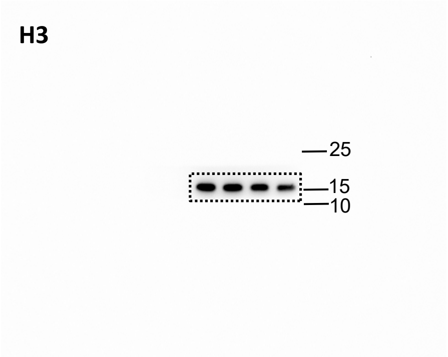
H3
25
15
10

Figure S-4G

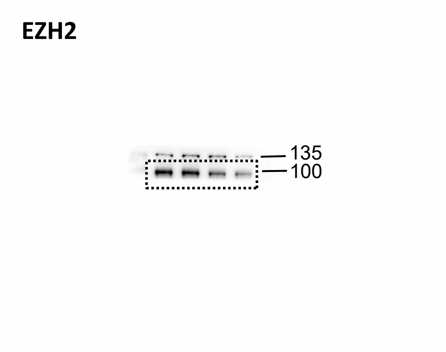
EZH2
135
100

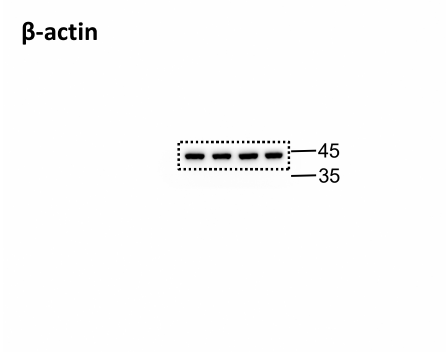
β-actin
45
35

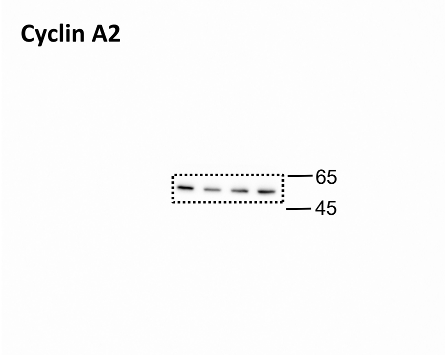
Cyclin A2
65
45

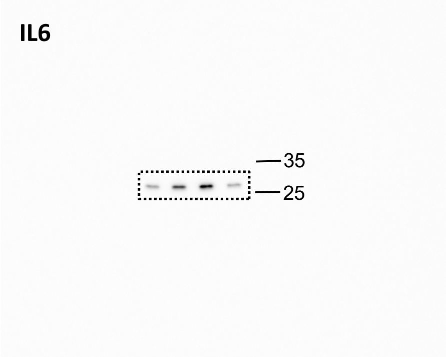
IL6
35
25

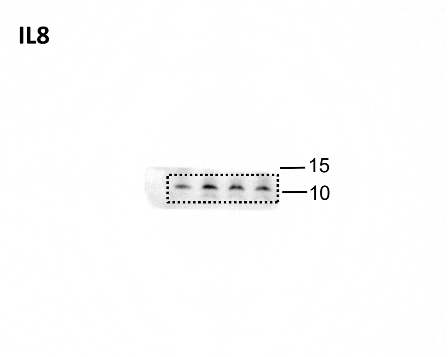
IL8
15
10

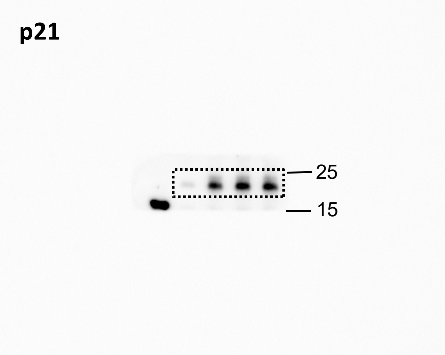
p21
25
15

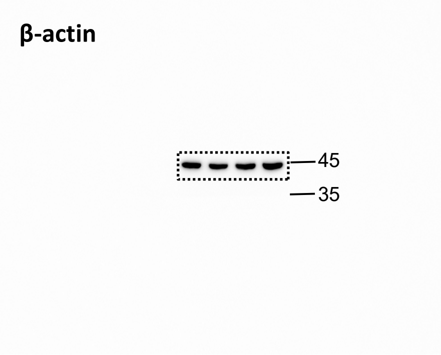
β-actin
45
35
